# Updating the Insecticide Resistance Status of *Aedes aegypti* and *Aedes albopictus* in Asia: A Systematic Review and Meta-Analysis

**DOI:** 10.3390/tropicalmed7100306

**Published:** 2022-10-17

**Authors:** Ramdan Zulfa, Wei-Cheng Lo, Po-Ching Cheng, Martini Martini, Ting-Wu Chuang

**Affiliations:** 1 International Master Program in Medicine, College of Medicine, Taipei Medical University, Taipei 11031, Taiwan; 2 Department of Molecular Parasitology and Tropical Diseases, School of Medicine, College of Medicine, Taipei Medical University, Taipei 11031, Taiwan; 3 Master Program in Applied Epidemiology, College of Public Health, Taipei Medical University, Taipei 11031, Taiwan; 4 Department of Epidemiology and Tropical Diseases, College of Public Health, Diponegoro University, Semarang City 50275, Indonesia

**Keywords:** *Aedes aegypti*, *Aedes albopictus*, insecticide resistance, meta-analysis, Asia

## Abstract

Background: *Aedes aegypti* and *Aedes albopictus* are two important vectors of several important arboviruses, including the dengue, chikungunya, and Zika viruses. Insecticide application is an important approach to reduce vector abundance during *Aedes spp.*-borne outbreaks in the absence of effective vaccines and treatments. However, insecticide overuse can result in the development of resistance, and careful monitoring of resistance markers is required. Methods: This meta-analysis and systematic review explored the spatial and temporal patterns of insecticide resistance in Asia from 2000 to 2021. PubMed, Scopus, EbscoHost, and Embase were used to enhance the search capability. The random-effects model was applied for the 94 studies that met our inclusion criteria for qualitative synthesis and meta-analysis. Results: Four major insecticides were studied (malathion, dichlorodiphenyltrichloroethane, permethrin, and deltamethrin). Dichlorodiphenyltrichloroethane resistance rates were high in both *Ae. aegypti* and *Ae. albopictus* (68% and 64%, respectively). Conversely, malathion resistance was less prevalent in *Ae. aegypti* (3%), and deltamethrin resistance was less common in *Ae. albopictus* (2%). *Ae. aegypti* displayed consistently high resistance rates (35%) throughout the study period, whereas the rate of insecticide resistance in *Ae. albopictus* increased from 5% to 12%. The rates of the major *kdr* mutations F1534C, V1016G, and S989P were 29%, 26%, and 22%, respectively. Conclusions: Insecticide resistance in both *Ae. aegypti* and *Ae. albopictus* is widespread in Asia, although the rates vary by country. Continuous monitoring of the resistance markers and modification of the control strategies will be important for preventing unexpected outbreaks. This systematic review and meta-analysis provided up-to-date information on insecticide resistance in dengue-endemic countries in Asia.

## 1. Introduction

Vector-borne diseases (VBDs) have become critical issues globally, being responsible for more than 700,000 deaths per year [1]. Dengue, chikungunya, and Zika are the major VBDs transmitted by *Aedes* mosquitoes, especially in tropical and subtropical regions [1]. These diseases are transmitted by *Aedes aegypti* and *Aedes albopictus* [2,3]. These vectors are prevalent in tropical and subtropical regions in Southeast Asia and Latin America [1]. Dengue has become endemic in 129 countries, and it is responsible for almost 390 million new infections annually [4,5,6,7]. The World Health Organization (WHO) reported that the incidence of dengue fever has increased eight-fold over the past two decades [8]. Chikungunya epidemics mainly occur in Latin America and South/Southeast Asia. Approximately 1.5 million people have contracted chikungunya in India, which has the most cases globally [9,10,11], and the disease has also spread to Indonesia, Maldives, Sri Lanka, Myanmar, and Thailand [12,13,14]. Since 2013, chikungunya outbreaks have been reported in Brazil, Bolivia, Colombia, Argentina, Cuba, Costa Rica, Ecuador, and Peru [10,15,16,17,18,19,20]. Zika outbreaks significantly impacted Latin America in 2016, causing more than 500,000 cases and nearly 2000 cases of microcephaly and/or central nervous system malformation in infants with congenital infection [21,22].

Dengue outbreaks place a significant burden on the population, economy, and health systems of the affected countries. Some countries in Asia have reported significant numbers of cases, including Bangladesh (101,000), Malaysia (131,000), the Philippines (420,000), and Vietnam (320,000) [8]. In addition, the first locally acquired cases of dengue fever in France and Spain were reported in October 2018 [23], and the recent re-emergence of dengue fever in the US [24] and Japan [25] indicates that the disease no longer has an exclusively tropical distribution.

Chikungunya virus infection can cause febrile sickness marked by severe and sometimes long-lasting polyarthritis. Unlike dengue, chikungunya is distinguished by persistent musculoskeletal disease typically affecting the peripheral joints that can last for months to years following acute infection [17,18,19,20]. Latin America and Southeast Asia have become major hotspots of chikungunya. In 2015, a total of 37,480 laboratory-confirmed cases of chikungunya were reported to the regional office of the Pan American Health Organization [26]. In 2019, India, Bangladesh, Pakistan, Sri Lanka, and the Maldives experienced large chikungunya outbreaks [27,28]. Since 2000, approximately 85% of chikungunya cases have occurred in South Asian countries [10,11,14,27,28,29,30,31,32].

Zika virus was first isolated from a sentinel rhesus monkey in Uganda in 1947 [33]. Before rapidly spreading across the Pacific Islands in the 21st century, Zika was documented in a small number of people in sub-Saharan Africa and then in Southeast Asia by the middle of the 20th century [34]. An unprecedented outbreak of Zika, which is linked to microcephaly and Guillain–Barré syndrome, was reported in Brazil and other Latin American countries in 2015 [35]. Zika was declared a public health emergency of international concern (PHEIC) by the WHO in February 2016 [36]. Cases of Zika have also been recently reported in several countries in Southeast Asia, including the Philippines [37], Vietnam [38], Indonesia [39,40,41], Malaysia [42], and Thailand [43].

The cocirculation of dengue, chikungunya, and Zika might have serious public health consequences for several reasons. First, the three diseases are transmitted by the same vectors, namely *Ae. aegypti* and *Ae. albopictus*, implying that their geographical distributions overlap. Second, the clinical symptoms of the diseases are similar, and misdiagnosis occurs if molecular assays are not used for diagnosis. Moreover, if a misdiagnosis were to occur, it might delay appropriate treatment or health care for severe dengue or result in brain/nervous system complications associated with Zika.

Currently, there are no effective treatments for dengue, and vaccines are under development. The one available vaccine, named Dengvaxia, has several limitations. Dengvaxia is recommended for people aged 6–45 years with previous dengue infection [44]. These limitations make it difficult to use Dengvaxia to prevent dengue transmission among vulnerable populations, such as young children and elderly persons, in dengue-endemic areas. Therefore, vector control (e.g., chemical control, biological control, source reduction, and public knowledge) is an important strategy to combat these mosquito-borne diseases. Source reduction is an extremely important strategy to reduce mosquito breeding sites through various habitat management approaches. Although community involvement in habitat management is critical, it is challenging to eliminate all breeding sites in the environment. During outbreak seasons, chemical control, such as the use of insecticides, is widely used to mitigate disease transmission quickly.

Well-managed insecticide spraying can reduce the abundance of mosquitoes effectively when outbreaks occur. There are four main classes of insecticides commonly used for vector control programs: pyrethroids, organophosphates (OPs), organochlorines (OCs), and carbamate.

OCs are chlorinated hydrocarbons that are frequently employed in the pest control industry. Insecticides belonging to this family include dieldrin, chlordane, chlorobenzoate, and dichlorodiphenyltrichloroethane (DDT). There are two subclasses of OC mechanisms: chlorinated insecticides (DDT type) and chlorinated alicyclic insecticides [45]. In mosquitoes, voltage-gated sodium channels (VGSCs) are the primary targets of DDT-type insecticides. DDT induces toxicity by maintaining the open state of sodium channels, prolonging the activation status, and gradually causing excitatory paralysis and mosquito death [46]. By contrast, chlorinated alicyclic insecticides bind to the gamma-aminobutyric acid (GABA) receptor, which is responsible for inhibiting neurotransmission, causing hyperexcitation of the nervous system [47,48].

OPs are derivatives of phosphoric acid. The most commonly used OPs are malathion, parathion, chlorpyrifos, and diazinon. The primary mechanism of OP insecticides is the inhibition of acetylcholinesterase (AChE), which can degrade acetylcholine into choline and acetic acid to halt nerve impulse transmission [49]. In the absence of AChE activity, acetylcholine continues to accumulate at the junction of the nerve cell and receptor site. Then, the continuation of nerve impulses results in the paralysis of a mosquito’s muscles and, eventually, death [50,51].

Pyrethroids are synthetic analogs of pyrethrin. Similar to DDT, type I pyrethroids (permethrin, tetramethrin, allethrin, and phenothrin) interfere with the function of VGSCs to prolong neurotransmission and paralyze mosquitoes [52,53,54]. Type-II pyrethroids that contain an α-cyano group (cyfluthrin, cyhalothrin, deltamethrin, and cypermethrin) induce choreoathetosis–salivation syndrome by modulating GABA receptors [52,54,55]. GABA binds GABA_A_ receptors on ligand-gated chloride ion channels, which control the chloride (Cl^−^) influx that maintains the membrane potential of neurons. The GABA_A_ receptor is the target of type-II pyrethroids. When the receptor is blocked by type-II pyrethroids, Cl^−^ influx and inhibitory functions are prevented, resulting in convulsions and death [56].

Carbamate insecticides are derivatives of carbamic acid. Carbamate insecticides, such as carbaryl, carbofuran, propoxur, and aldicarb, exhibit effects similar to OPs by inhibiting AChE activity [57]. Unlike OPs, carbamates can be rapidly metabolized by mosquitoes [58].

The major challenge of utilizing chemical control is the emergence of insecticide resistance in the targeted populations. Insecticide resistance can be divided into four main mechanisms. Metabolic resistance involves elevated levels or activities of esterases, which metabolize or degrade insecticides before their toxic effects appear [59]. Esterase, monooxygenases, and glutathione S-transferases are the three main enzyme systems responsible for metabolic resistance [60,61]. Target site resistance refers to the genetic modification of structures targeted by certain insecticides. Resistance caused by multiple mutations in the knockdown resistance (*kdr*) gene on VGSCs has been studied for pyrethroids and DDT [62]. Point mutations in *kdr* might change the structure of specific binding sites to reduce the binding efficiency of certain insecticides [56]. In the reduced penetration mechanism, mosquito cuticles are modified to prevent or slow the absorption of insecticides [63]. Behavioral resistance refers to the behavioral response of mosquitoes that avoid contact with insecticides.

Growing urbanization and the frequent movements of people, along with climate changes, might accelerate the propagation of *Ae. aegypti* and *Ae. albopictus* and cause unexpected outbreaks in Asia [64,65,66,67]. The application of large-scale insecticide spray to control more frequent outbreaks can be expected, and the risk of emerging insecticide resistance has also been raised in Asia. Insecticide resistance has become an important public health issue that could jeopardize vector control and disease prevention. Moyes et al. reviewed multiple datasets of insecticide resistance in *Ae. aegypti* and *Ae. albopictus* from 2008 to 2015 and revealed that the patterns of resistance to two commonly used insecticides, deltamethrin for *Ae. aegypti* and temephos for *Ae. albopictus*, varied by geographical area [68]. Widespread insecticide resistance has been reported in several Asian countries, including Thailand, India, Malaysia, China, Vietnam, and Indonesia, which have a high burden of mosquito-borne diseases [69,70,71,72,73,74,75,76,77]. These studies were conducted in different settings, and they lack an integrated approach to summarizing the overall patterns of insecticide resistance in Asia. Thus, providing updated and comprehensive information on insecticide resistance in Asia is critical for insecticide sensitivity monitoring and vector control strategies. This study conducted a meta-analysis to summarize the up-to-date information on insecticide resistance in *Ae. aegypti* and *Ae. albopictus* in the Asia region from 2000 to 2021. These data will help governments and public health policymakers understand the current insecticide resistance status and modify vector control strategies accordingly.

## 2. Materials and Methods

### 2.1. Study Design and Search Strategy

This systematic review and meta-analysis followed the Preferred Reporting Items for Systematic Reviews and Meta-analysis (PRISMA) statement [78]. The study has been registered on PROSPERO (CRD42022291803) and reported following the Meta-analysis of Observational Research in Epidemiology guidelines [79]. We reviewed the existing published studies that reported the prevalence of insecticide resistance in *Ae. aegypti* and *Ae. albopictus*. Four databases, namely, PubMed, Scopus, EbscoHost, and Embase, were used to enhance the search capability. The search keywords were established by combining “*Aedes aegypti*” OR “*Aedes albopictus*”, “Organophosphate” OR “Organochlorine” OR “Pyrethroid,” AND “Resistance” The details of the search strategy are provided in Table S1.

### 2.2. Study Eligibility

The articles were extracted from the aforementioned databases using the aforementioned keywords. Studies utilizing insecticide bioassays and examining *kdr* mutations in *Ae. aegypti* or *Ae. albopictus* were included in the literature review. In addition, we limited the study region to Asia and the study period to 2000–2021. Studies based on laboratory-maintained rather than field-maintained treatment populations, those lacking quantitative data, and those focusing on behavior or biology/ecology were excluded. The detailed information of the eligible articles is presented in Table S2.

### 2.3. Data Extraction and Quality Assessment

Two reviewers separately extracted the data, and disagreements were resolved via discussion. The insecticide resistance rate was extracted or recalculated from eligible articles. The following variables were included in the analysis: first author’s name and publication year, study period, country, bioassay method, insecticide type, insecticide class, and *kdr* mutation site.

We utilized the checklist created by Hoy et al. (2012) to evaluate the consistency of each study’s reporting and the risk of bias [78]. This instrument contains 10 items, including 4 items assessing external validity and 6 items assessing internal validity. The items are formulated as binary questions (yes/no). Low, moderate, and high risks of bias are indicated by scores of 9–10, 7–8, and ≤6, respectively [80]. The risk of bias was moderate in 43 studies and low in 51 studies (Table S3).

### 2.4. Data Analysis

In total, 28 insecticide types within 5 classes were tested in this study (Figure S1). Malathion (OP), DDT (OC), permethrin (Pyrethroid), and deltamethrin (pyrethroid) are the four major types of insecticides used in Asia and the number of studies for the four types of insecticides are 40, 34, 50, and 45, respectively (Figure S1). Thus, the 4 types of insecticide were selected in the meta-analysis. The study outcome was the determination of the insecticide resistance prevalence rates of each insecticide in *Ae. aegypti* and *Ae. albopictus*. We also analyzed three prevalent *kdr* mutations (F1534C, V1016G, and S989P) in *Ae. aegypti*. The *kdr* analysis was not performed in *Ae. albopictus* because of the insufficient sample size. Random-effects models were applied to estimate the prevalence with a 95% confidence interval [81]. The between-study heterogeneity was evaluated using the *I*^2^ statistic. To identify the possible sources of heterogeneity, we evaluated country, human development index (HDI), and study quality as variables in the meta-regression models. Study quality refers to the risk of bias. Lastly, visual inspection of the Doi plot and the Luis Furuya–Kanamori index (LFK index) was used to assess publication bias [82]. An asymmetrical Doi plot indicates the existence of publication bias. An LFK index of −1 to 1 indicates a symmetrical plot, an index within ±2 indicates minor asymmetry, and an index of <−2 or >2 indicates major asymmetry [83].

The meta-analysis was performed using the “metaprop” package in R (version R.3.6.3 Foundation for Statistical Computing). The spatial distributions of insecticide resistance in Asia were visualized using the geographic information system technique. The resistance maps were produced by ArcGIS Pro 2.9 (ESRI, Redlands, CA, USA).

## 3. Results

### 3.1. Literature Survey

**Figure 1:**
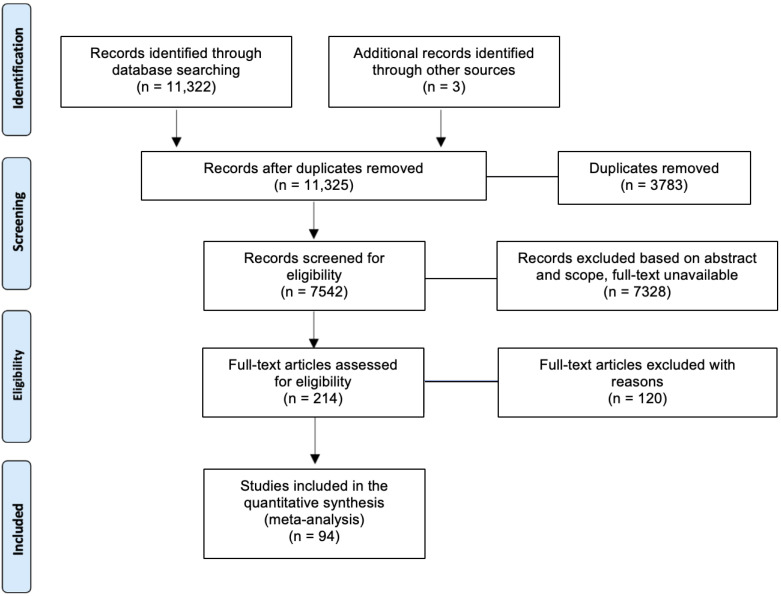
PRISMA flow diagram of article selection.

In total, 11,325 publications were extracted from the databases (Figure 1). Three additional articles were recruited from other sources. After removing duplicates and unqualified articles, 94 articles met our inclusion criteria for qualitative synthesis and meta-analysis.

**Figure 2:**
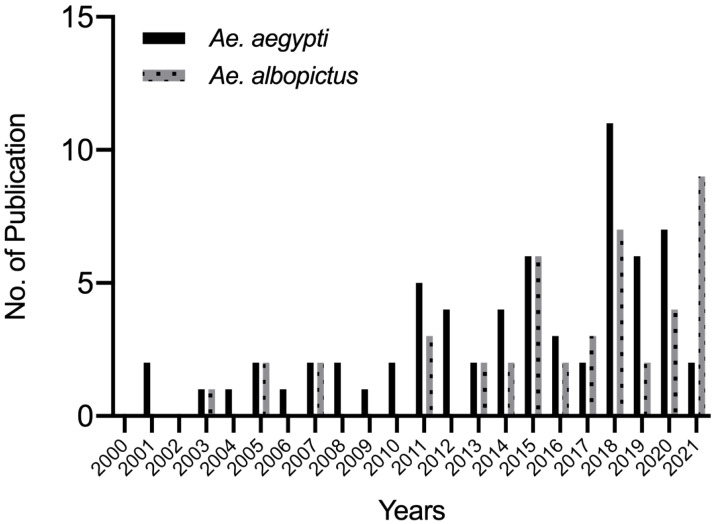
Temporal patterns of publications.

Figure 2 presents the temporal trends of the insecticide resistance studies of *Ae. aegypti* and *Ae. albopictus* from 2000 to 2021. Approximately 50 articles examined *Ae. aegypti,* 18 articles tested *Ae. albopictus*, and 17 articles examined both species. The nine remaining articles only examined the *kdr* mutation sites. The number of articles on *Ae. aegypti* gradually increased over time and peaked in 2018. Meanwhile, studies on *Ae. albopictus* were sparse until 2010 before significantly increasing.

**Figure 3:**
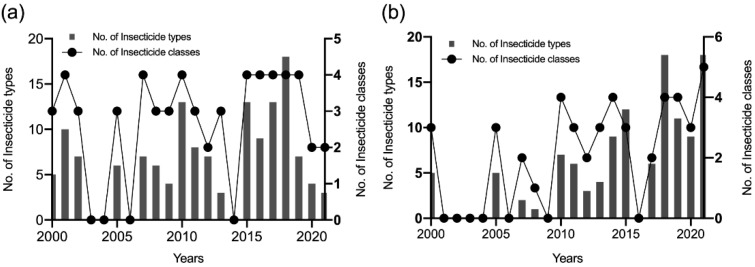
Types and classes of insecticides tested in *Aedes* mosquitoes. (**a**) *Ae. aegypti*. (**b**) *Ae. albopictus*.

Figure 3 presents the number of insecticide types and classes tested in *Ae. aegypti* and *Ae. albopictus* in 2000–2021. Overall, more than 10 insecticide types and 5 classes were tested in both mosquito species after 2015.

### 3.2. Spatial Patterns of Insecticide Resistance in Asia

**Figure 4:**
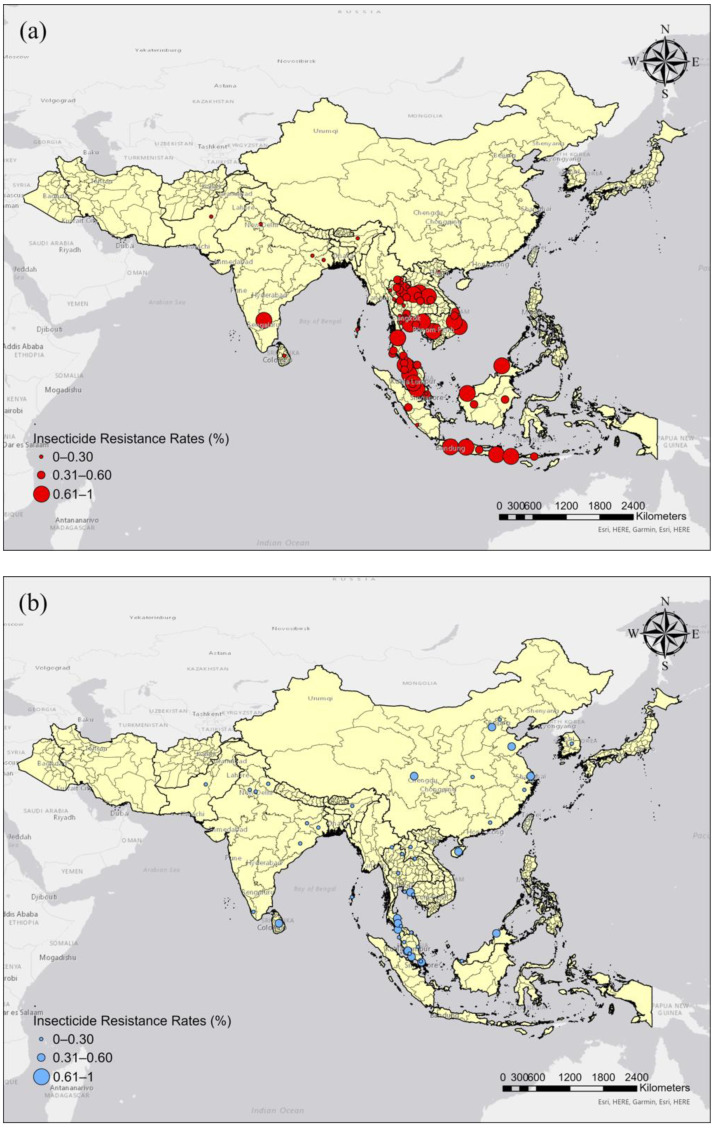
Spatial distribution of insecticide resistance in *Aedes* mosquitoes. (**a**) *Ae. aegypti*. (**b**) *Ae. albopictus.* The sizes of the circles correspond to the insecticide resistance rates.

The spatial distribution of insecticide resistance in *Ae. aegypti* and *Ae. albopictus* is presented in Figure 4. *Ae. aegypti* exhibited higher insecticide resistance rates than *Ae albopictus*, especially in Southeast Asia (Figure 4a). Studies on *Ae. aegypti* were mainly conducted in Thailand, Malaysia, Indonesia, Singapore, Vietnam, Laos, Cambodia, India, and Pakistan. Studies on *Ae. albopictus* were conducted in Malaysia, Thailand, China, India, Sri Lanka, South Korea, and Pakistan (Figure 4b).

### 3.3. Spatial Patterns of Resistance to Malathion, DDT, Permethrin, and Deltamethrin

**Figure 5:**
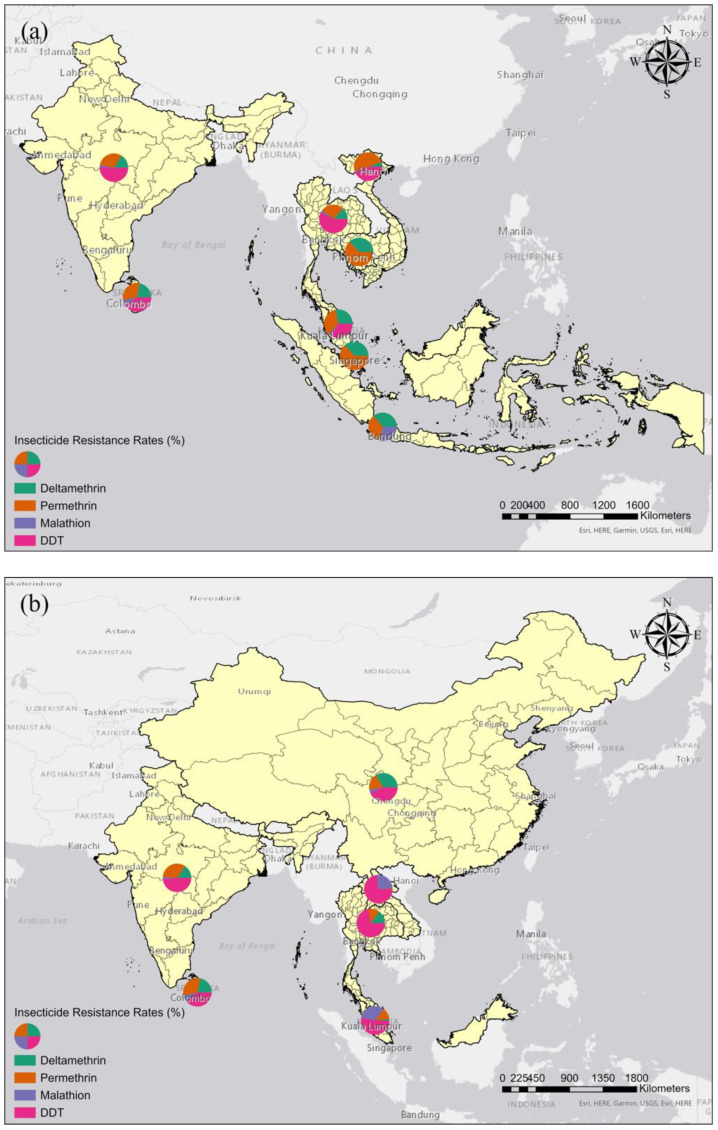
Resistance rates for the four major insecticides in each country/region. (**a**) *Ae. aegypti.* (**b**) *Ae. albopictus*.

The country-specific resistance rates of four major insecticides are presented in Figure 5. *Ae. aegypti* displayed high resistance to deltamethrin (69%), permethrin (70%), and malathion (54%) in Indonesia (Figure 5a). *Ae. aegypti* exhibited high resistance to DDT (94%), permethrin (43%), and deltamethrin (21%) in Thailand (Figure 5a), whereas high resistance to DDT was observed in *Ae. albopictus* (30%) (Figure 5a,b). In Malaysia, *Ae. aegypti* displayed high resistance to DDT (42%), permethrin (44%), and deltamethrin (36%) (Figure 5a). In addition, *Ae. albopictus* also displayed high resistance to DDT (62%) and malathion (38%). *Ae. aegypti* exhibited high resistance to permethrin (99%) and deltamethrin (58%) in Cambodia (Figure 5a). *Ae. aegypti* in Singapore exhibited high resistance to permethrin (89%) and deltamethrin (51%) (Figure 5a). *Ae. aegypti* in Vietnam displayed high resistance to DDT (81%) and permethrin (93%) (Figure 5a). *Ae. albopictus* exhibited high resistance to DDT (58%) in Laos (Figure 5b). *Ae. albopictus* displayed high resistance to DDT (34%) in China (Figure 5b). In South Asia, *Ae. aegypti* and *Ae. albopictus* mainly exhibited resistance to DDT, permethrin, and deltamethrin.

### 3.4. Prevalence of Insecticide Resistance

**Figure 6:**
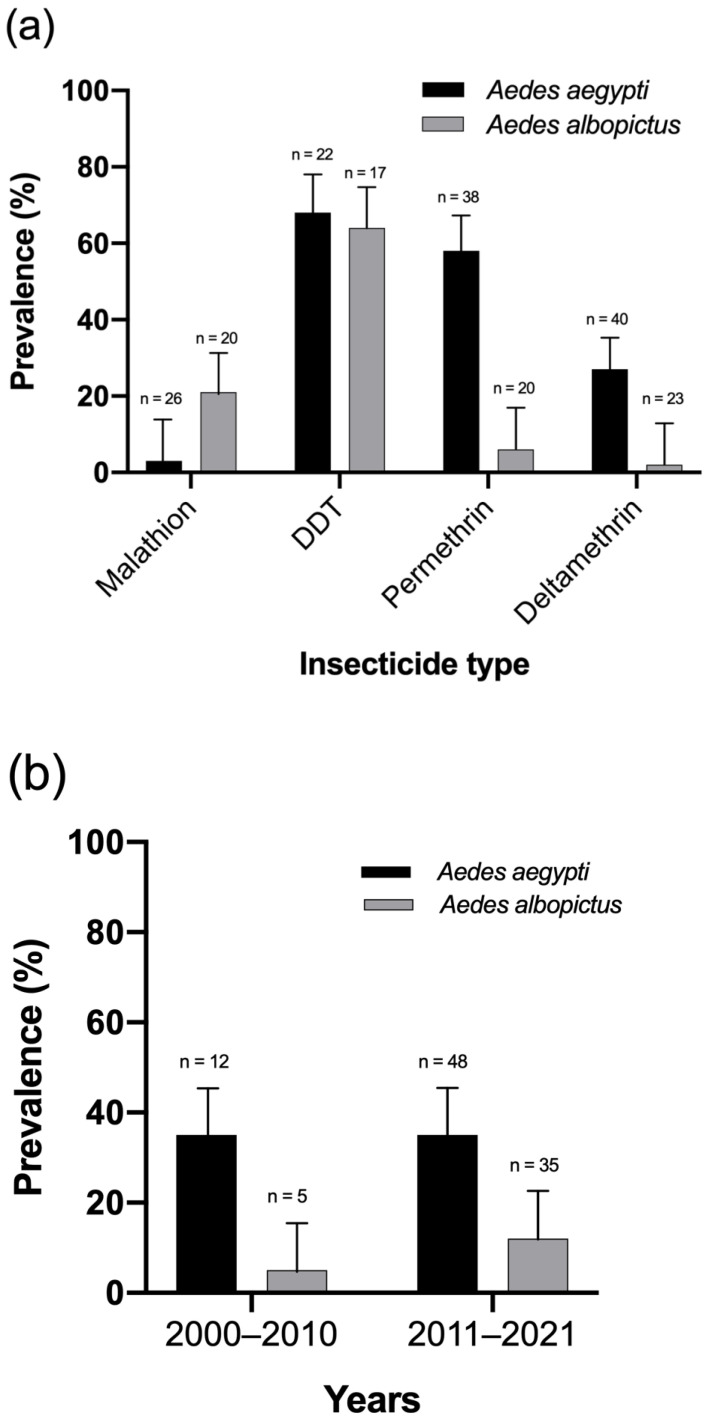
(**a**) Meta-analysis results of the summarized insecticide resistance rates. (**b**) Subgroup meta-analysis of insecticide resistance rates for the two time periods.

The summarized meta-analysis results of the four major insecticides are presented in Figure 6a. The comprehensive forest plots for the major insecticides on *Ae aegypti* and *Ae. albopictus* are presented in Figure S2A–H. The overall prevalence of insecticide resistance to *Ae. aegypti* was 3% for malathion, 68% for DDT, 58% for permethrin, and 27% for deltamethrin. On the other hand, the prevalence of insecticide resistance to *Ae. albopictus* was 21% for malathion, 64% for DDT, 6% for permethrin, and 2% for deltamethrin. A subgroup analysis examined the temporal patterns of insecticide resistance by dividing the published articles into two groups (2000–2010 vs. 2011–2021; Figure 6b), and an increasing prevalence of insecticide resistance was observed in *Ae. albopictus* (5% vs. 12%, respectively; Figure 6b, Figure S2I–L).

**Figure 7:**
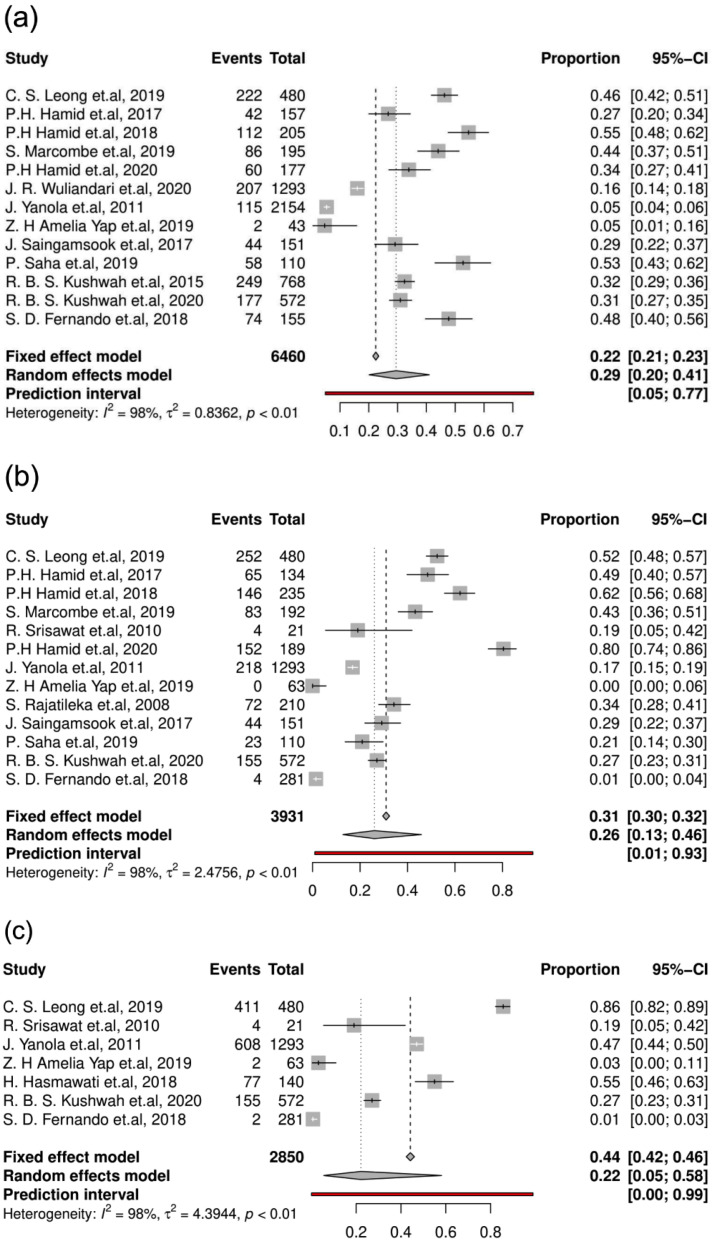
Forest plots of *kdr* mutations in *Ae.* *Aegypti*. (**a**) F1534C; (**b**) V1016G; and (**c**) S989P. Events = the number of insecticide-resistant mosquitoes; total = the total number of mosquitoes tested.

*Ae. aegypti* featured seven *kdr* mutations in six codons in VGSC (Table S4), and F1534C, V1016G, and S989P were the major mutations. The F1534C, V1016G, and S989P mutation rates in *Ae. aegypti* were 29%, 26%, and 22%, respectively (Figure 7).

### 3.5. Heterogeneity and Publication Bias

Substantial heterogeneity was observed in this analysis, and therefore, meta-regression analysis was performed to evaluate whether insecticide resistance was influenced by the country, HDI, and study quality. Significant country effects were observed in the meta-regression model for *Ae. aegypti* (*p* = 0.0014). Using Thailand as the reference country, Malaysia and Vietnam displayed positive country effects (Tables S5 and S6). Country effects were also detected (*p* = 0.0237) for *Ae. albopictus*. The HDI only exhibited a statistically significant effect (*p* = 0.0121) for *Ae. aegypti*, as a higher HDI increased the insecticide resistance rate (Tables S7 and S8). The development level also affected insecticide resistance rates in *Ae. aegypti*. The study quality exerted a nonsignificant effect on insecticide resistance in both *Ae. aegypti* and *Ae. albopictus* (Tables S9 and S10).

Publication bias was evaluated using the Doi plot and LFK index. The Doi plot of *Ae. aegypti* featured minor asymmetry (LFK index = 1.34; Figure S3a), whereas major asymmetry was noted for *Ae. albopictus* (LFK index = 5.23; Figure S3b). The Doi plots and LFK index indicated that studies recording high insecticide resistance rates are more likely to be published.

## 4. Discussion

Our study summarized insecticide resistance patterns in both *Ae. aegypti* and *Ae. albopictus* in Asian countries, primarily focusing on malathion, DDT, permethrin, and deltamethrin, the most commonly used insecticides for vector control. In summary, malathion is the most effective insecticide against *Ae. aegypti* in Asia, whereas permethrin and deltamethrin remain useful for controlling *Ae. albopictus*.

The present results echoed previous studies in Thailand that reported high rates of deltamethrin and permethrin resistance in *Ae. aegypti* [74,75,77]. Conversely, the results suggested that permethrin and deltamethrin remain effective against *Ae. albopictus* in Malaysia. Meanwhile, the widespread use of deltamethrin and permethrin as adulticides in place of DDT could explain the emergence of insecticide resistance in Indonesia. Furthermore, the extensive resistance of *Ae. aegypti* to malathion in Indonesia suggested that policies supporting the widespread use of malathion fogging to control *Ae. aegypti* require further evaluation [68]. Similar to the findings in other Southeast Asian countries, *Ae. aegypti* in Singapore and Cambodia was strongly resistant to permethrin and deltamethrin, whereas malathion has retained efficacy for vector control. Moderate resistance to DTT and low-to-moderate resistance to permethrin and deltamethrin were observed for both *Ae. aegypti* and *Ae. albopictus* in South Asia.

The recent emergence of COVID-19 and the resulting lockdown policies might have contributed to an increased prevalence of dengue in Asia. Thailand, Malaysia, and Singapore implemented lockdown policies to prevent the spread of COVID-19. Thailand experienced a dengue outbreak during a lockdown for COVID-19 [84]. According to a follow-up study, the dengue prevalence in Singapore increased by approximately 37.2% among working adults because of the lockdown policy [85]. The recent dengue outbreak during the COVID-19 outbreak might have increased intensive vector control and potentially enhanced the emergence of insecticide resistance in those countries.

A previous study revealed an uncommon mutation (I1532T) in *Ae. albopictus* in China [86]. Another study stated that the I1532T variant has only been found in *Ae. albopictus* in Italy. In addition, mutations in codons 989 and 1016, which are normally found in *Ae. aegypti*, were also found in *Ae. albopictus* in Asia [68]. This finding suggests that the same mutations might arise in both species.

F1534C is the most common point mutation in *kdr* in *Ae. aegypti*. This mutation confers resistance to permethrin and deltamethrin when combined with other mutations [87,88]. The V1016G mutation is commonly found in Asia, whereas V1016I is commonly found in the Americas [68]. In some cases, coincident V1016G and S989P mutations have been found to cause a higher level of resistance [87]. Triple-mutant haplotypes (989P, 1016, and 1534C) were found in a previous study, implying the evolution of higher resistance [68]. Future studies to understand *kdr* mutation sites relevant to phenotypic effects are critical in Asia.

The efficiency of disease control will be jeopardized if the resistant strains become dominant in the vector populations. Furthermore, insecticides can have a significant impact on the environment and on ecosystems [89]. Alternative control measures to reduce mosquito abundance and disease transmission are being assessed in the field [90]. Genetically engineered *Ae. aegypti* infected with *Wolbachia* is a promising approach to control dengue transmission that has been tested in many countries [91]. Utarini et al. demonstrated that the *Ae. aegypti wMel* strain successfully reduced symptomatic dengue and hospitalization in a randomized controlled trial in Yogyakarta, Indonesia [92]. Similar evidence has been reported in Brazil and Australia [93,94,95]. Despite the successful implementation of *Wolbachia*-infected mosquitoes into communities, the impact of insecticide usage and *Wolbachia*-infected vectors is unknown. Tantowijoyo et al. compared mosquito abundance and insecticide resistance between the *Wolbachia*-treated and control areas in Yogyakarta. The results revealed similar insecticide resistance rates between the two groups, and the researchers concluded that insecticide resistance might not confound the *Wolbachia* trial [96]. At the current stage, both the novel control strategy and insecticide treatment are being implemented in a parallel manner. Thus, monitoring insecticide resistance remains critical for disease control.

This study applied meta-analysis to reveal updated insecticide resistance patterns in Asia. However, our study had some limitations. First, there is no standardized protocol or system to monitor insecticide resistance in different Asian countries. The effort to test or report resistance varied by region, and thus, reporting bias was unavoidable in our analysis. The heterogeneity and meta-regression models also reflected these issues. Second, we only included articles written in English. Collaboration with local public health workers might be important to access resistance surveys written in other languages in the future to improve the diversity of the analysis. Third, although we observed statistical evidence of publication bias in both *Ae. aegypti* and *Ae. albopictus* populations, the bias was stronger for *Ae. albopictus*. Additional studies focusing on *Ae. albopictus* are required in the future to reduce the bias and enhance knowledge regarding insecticide resistance in secondary, but more widely distributed, vectors of dengue, Zika, and chikungunya.

In summary, this study has revealed some types of insecticides that are still effective for controlling *Ae. aegypti* and *Ae. albopictus* in Asia; however, the standardized protocols for monitoring insecticide resistance should be developed and administered by public health sectors in different countries in Asia. Timely information is critical for public health workers to modify vector control strategies to prevent outbreaks

## 5. Conclusions

Insecticide resistance, especially DDT resistance, is widely distributed in both *Ae. aegypti* and *Ae. albopictus* in Asia. *Ae. aegypti* also displayed moderate resistance to deltamethrin and permethrin. Malathion remains effective for *Ae. aegypti* control, whereas deltamethrin and permethrin are effective against *Ae. albopictus.* Moderate rates of F1534F, V1016G, and S989P mutations in *kdr* were detected in *Ae. aegypti*. The country and HDI had different effects on the heterogeneity of the analysis, indicating that insecticide resistance in Asia is highly variable with respect to space, time, and socioeconomic factors.

Asian countries are major hotspots for dengue, Zika, and chikungunya. The emergence of insecticide resistance is a critical public health issue. Integrated vector control strategies combined with new techniques might effectively reduce the transmission of mosquito-borne disease. This study provided updated and comprehensive information on insecticide resistance in *Ae. aegypti* and *Ae. albopictus* in Asia. This information can help public health authorities in different countries to modify their control strategies and prevent future outbreaks.

## Data Availability

Not applicable.

## Supplementary Materials

The following supporting information can be downloaded at https://www.mdpi.com/article/10.3390/tropicalmed7100306/s1, Figure S1: The frequency of insecticide resistance tested in the eligible articles; Figure S2: Forest Plots of meta-analysis; Figure S3: Publication bias Doi plot and LFK index; Table S1: Searching string details; Table S2: Characteristics of studies included in the systematic review; Table S3: Risk of Bias (RoB) assessment; Table S4: Knockdown resistance (*kdr*) mutations that have been detected in different mosquito populations in Asia Region; Tables S5–S10: Meta-regression results. References [97,98,99,100,101,102,103,104,105,106,107,108,109,110,111,112,113] are cited in the supplementary materials.
