# Supplementary material for: Updating the Insecticide Resistance Status of Aedes aegypti and Aedes albopictus in Asia: A Systematic Review and Meta-Analysis
Source: Trop Med Infect Dis. 2022 Oct 17;7(10):306. doi: 10.3390/tropicalmed7100306 (PMC9607256; doi:10.3390/tropicalmed7100306)
Supplement: Supplementary file 1 [file tropicalmed-07-00306-s001.zip › Figure S2.pdf]

A. Malathion (*Aedes aegypti*)

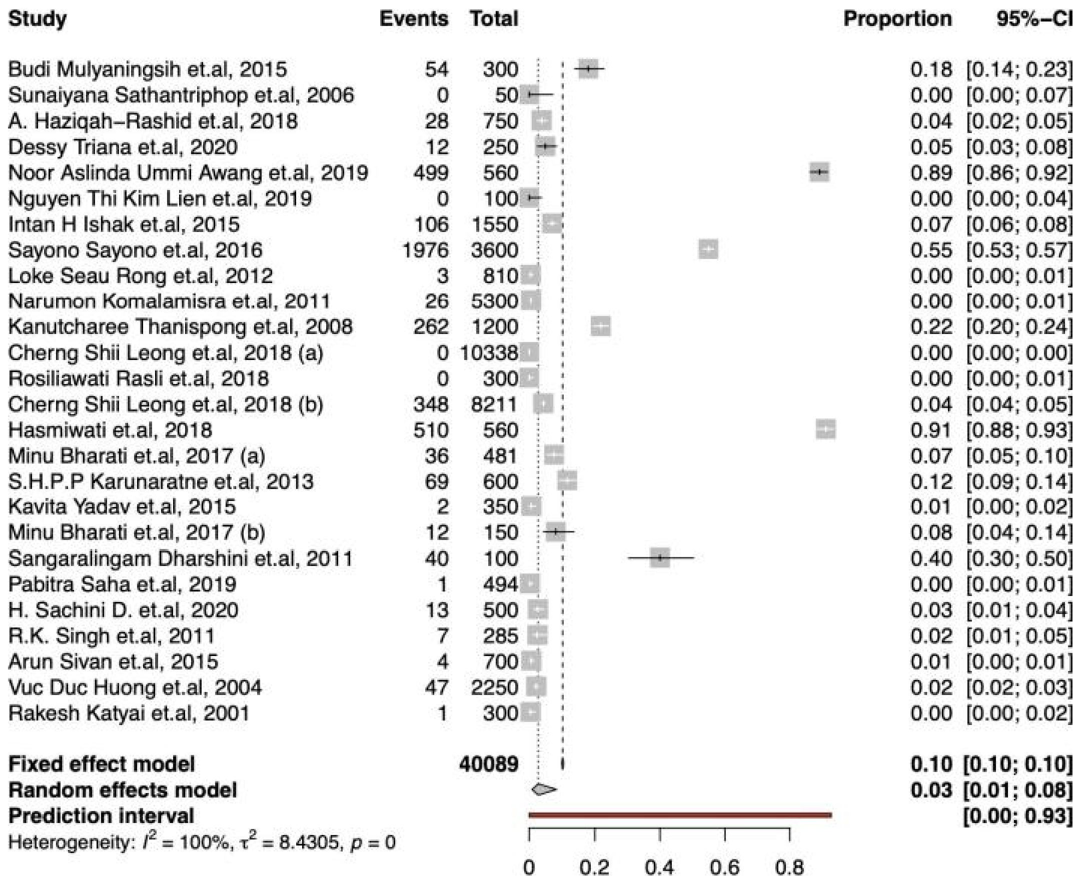

B. DDT (*Aedes aegypti*)

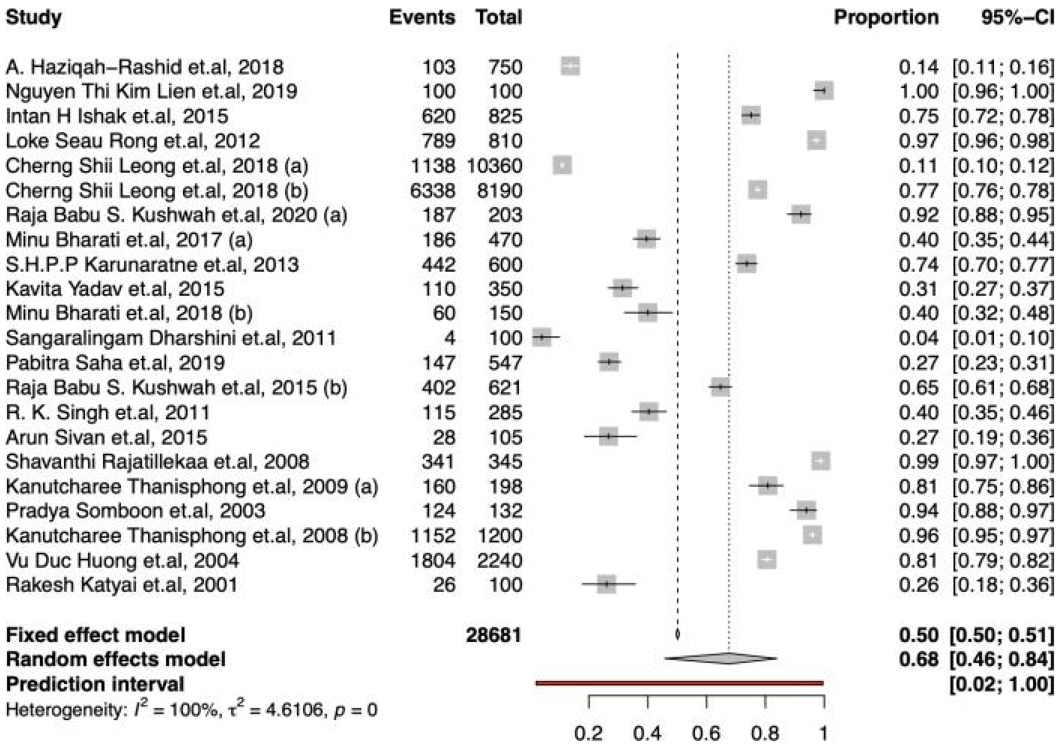

C. Permethrin (*Aedes aegypti*)

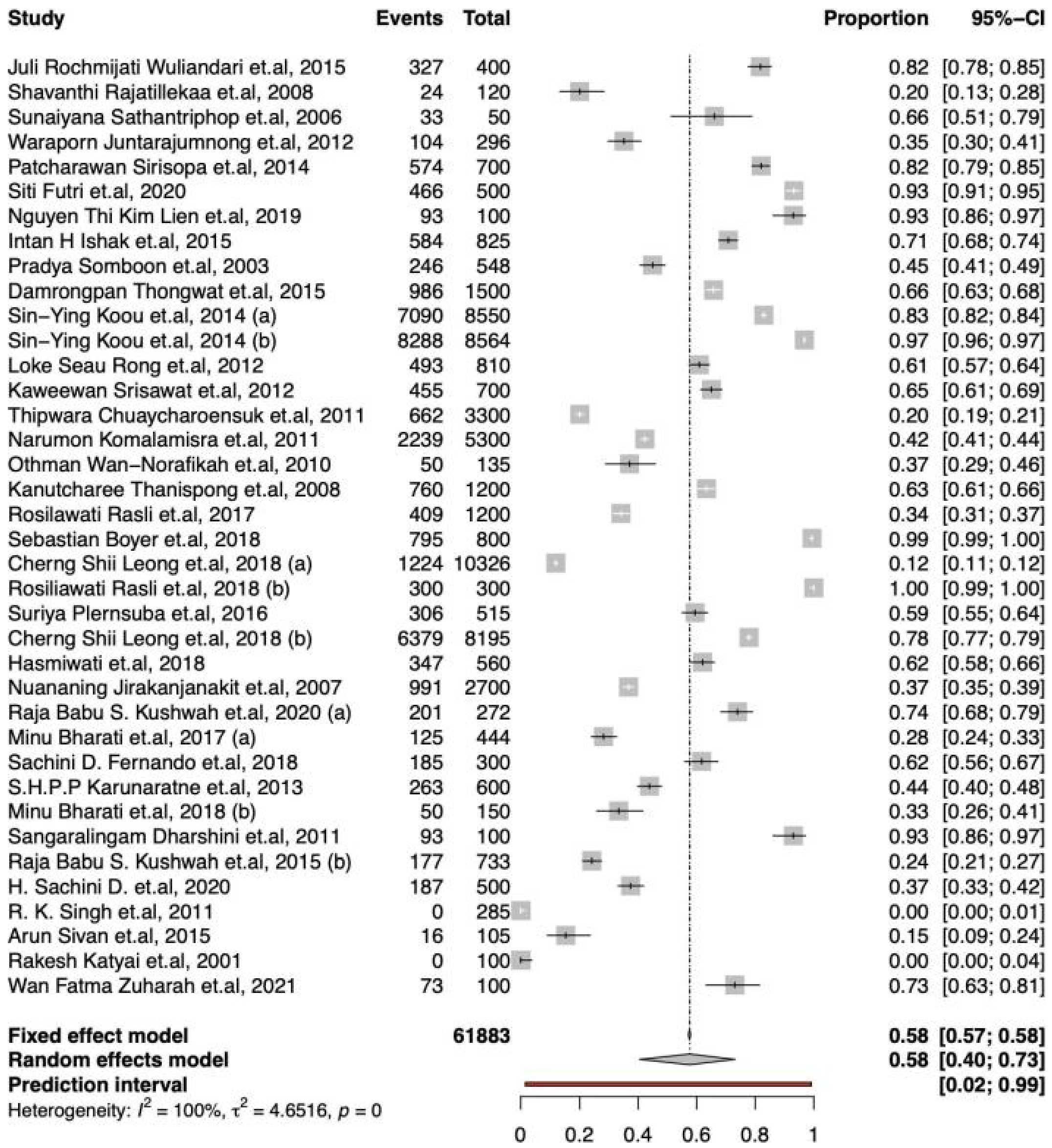

D. Deltamethrin (*Aedes aegypti*)

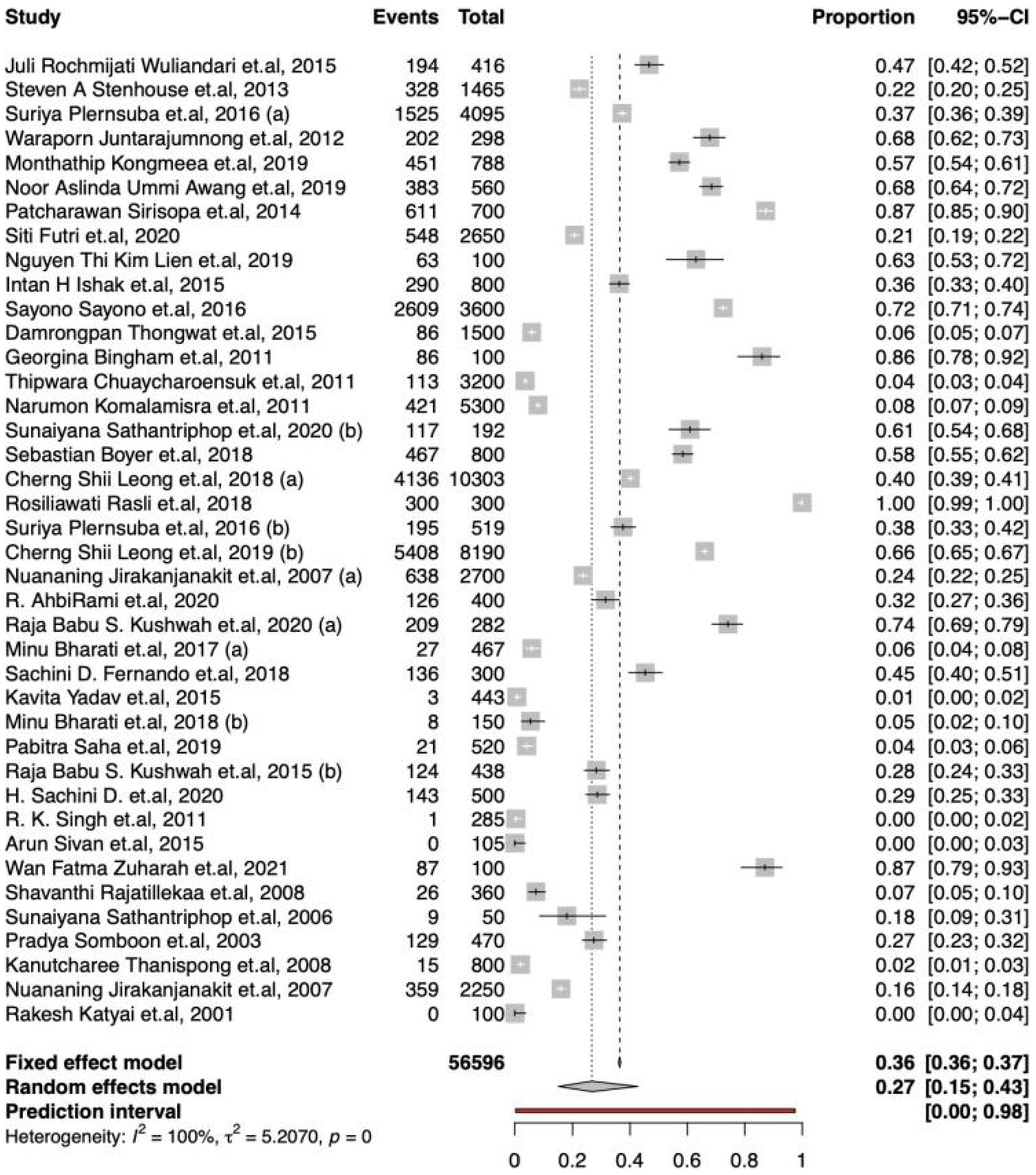

E. Malathion (*Aedes albopictus*)

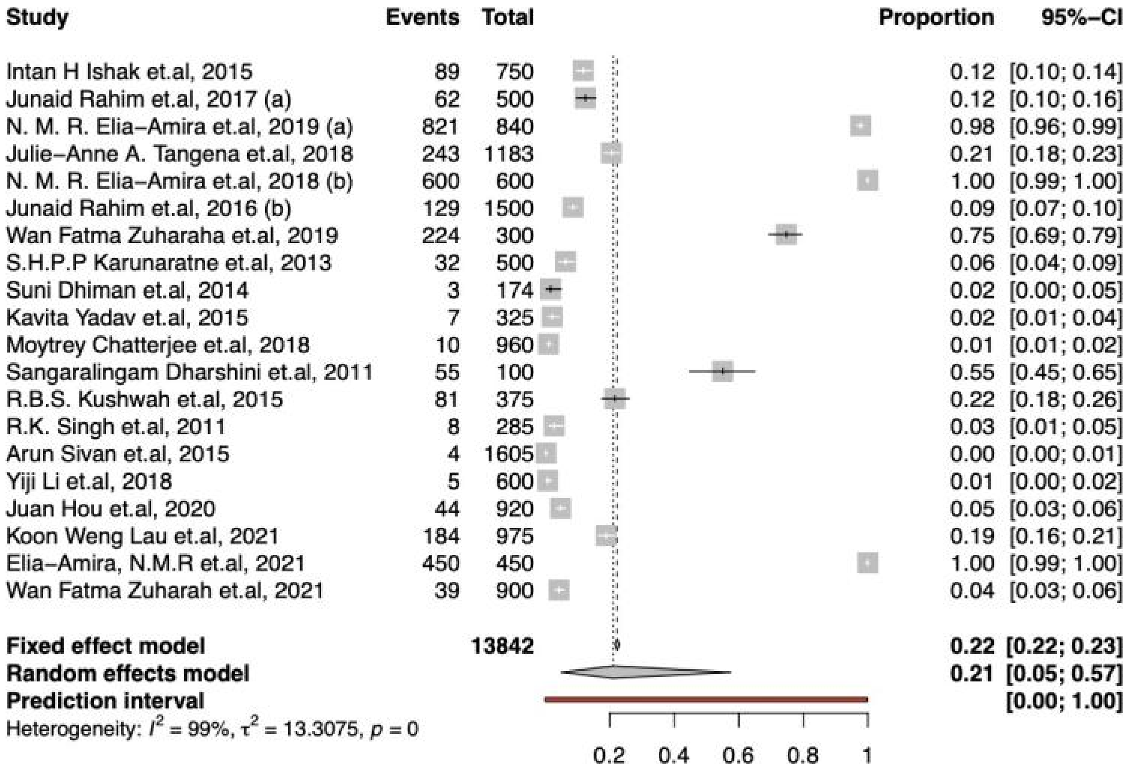

F. **DDT (*Aedes albopictus*)**

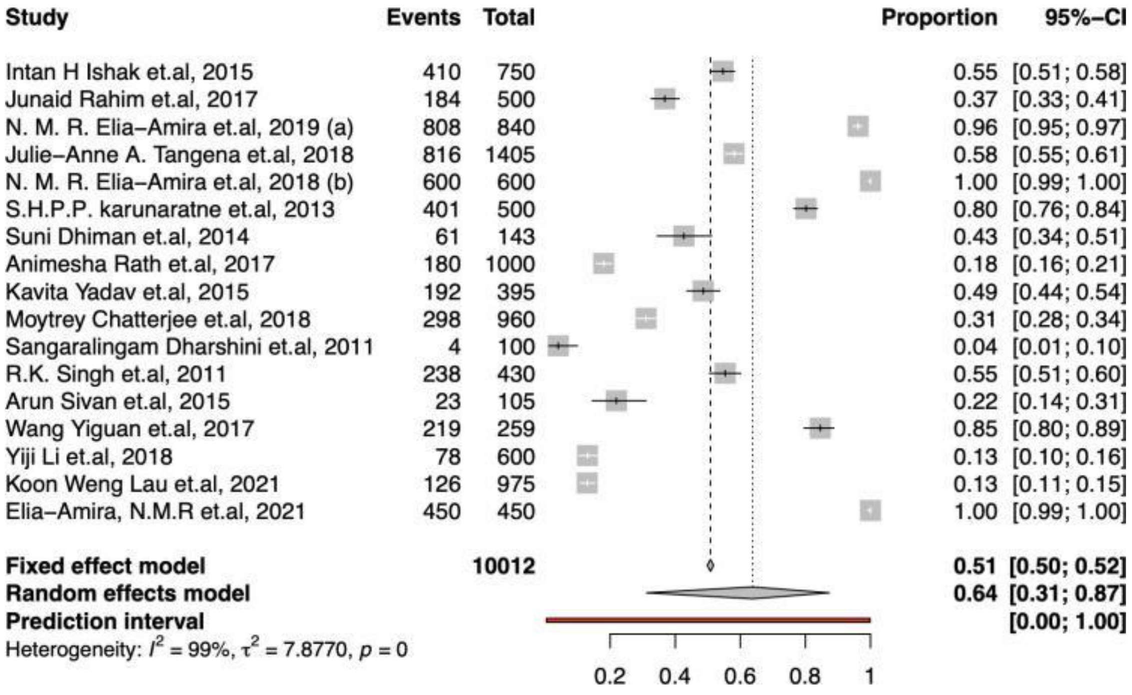

G. **Permethrin (*Aedes albopictus*)**

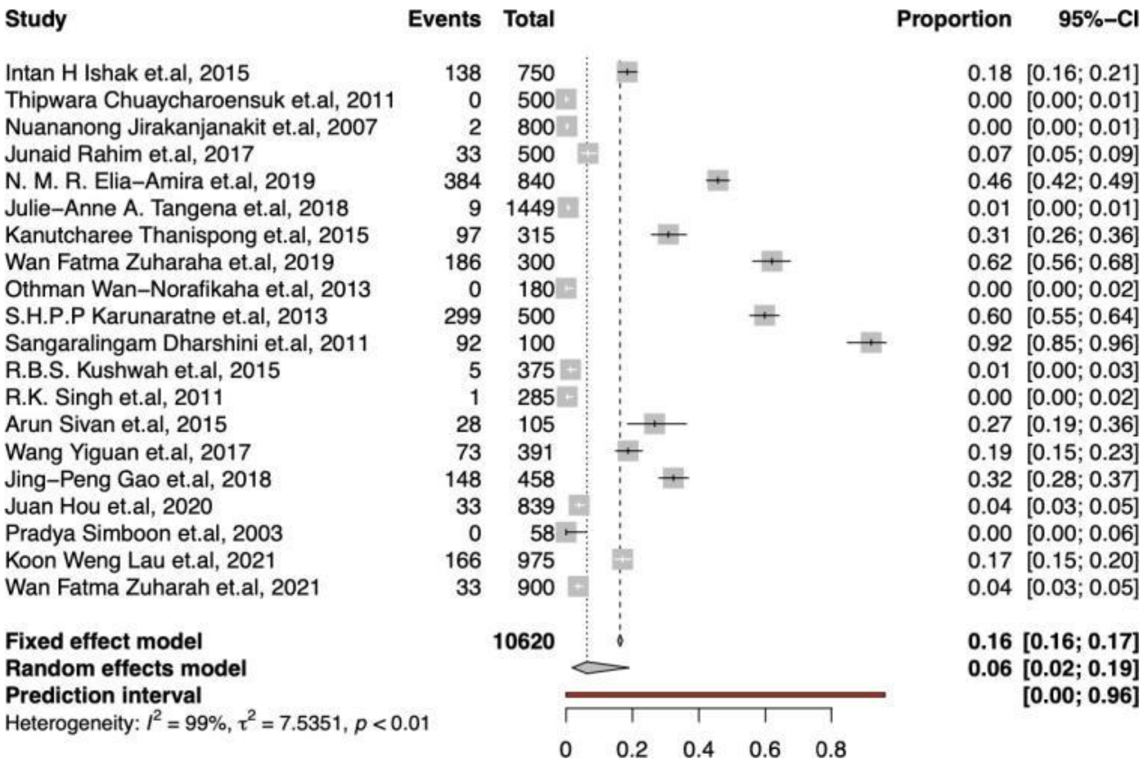

H. Deltamethrin (*Aedes albopictus*)

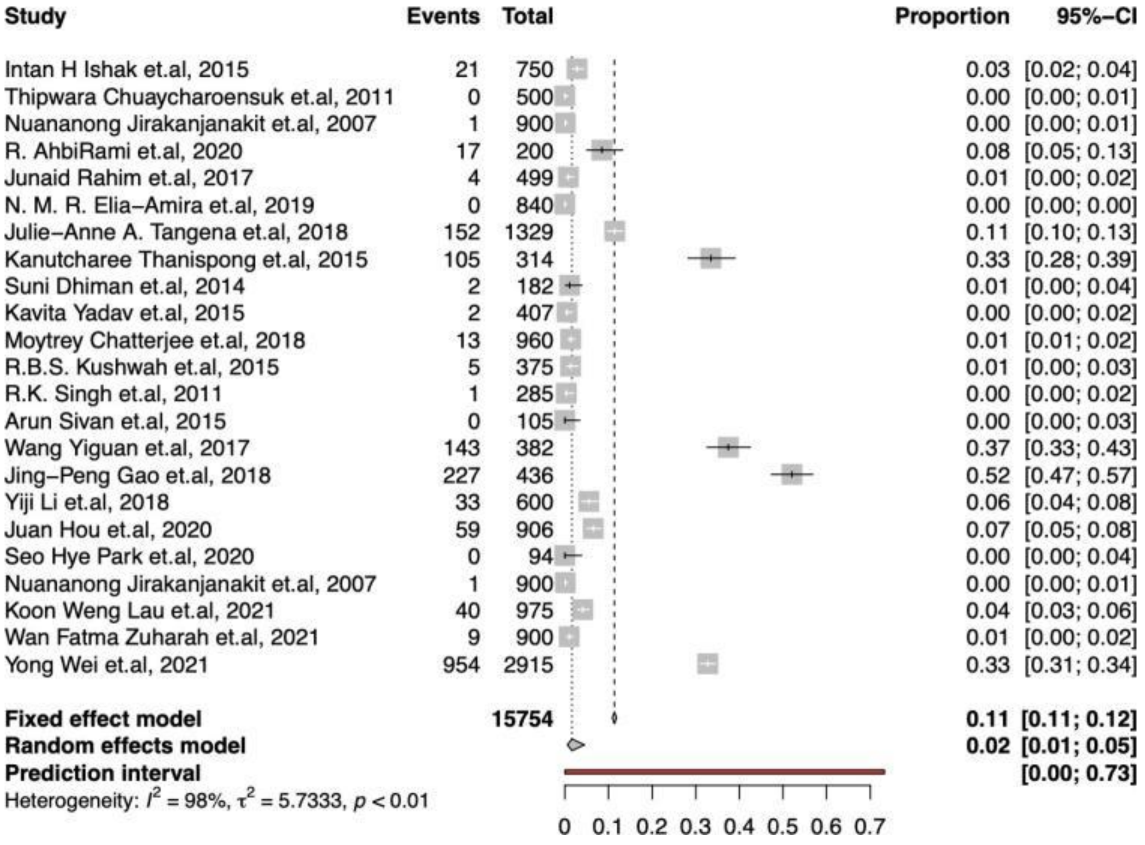

I. 2000-2010 (*Aedes aegypti*) Sub-group Analysis

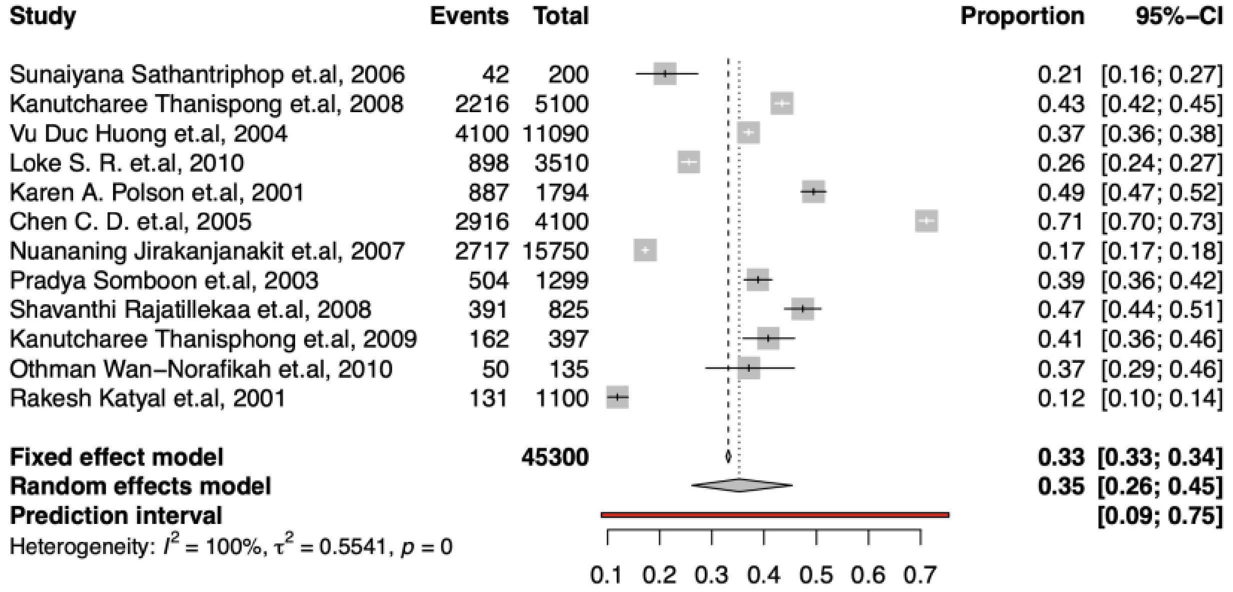

J. 2011-2021 (*Aedes aegypti*)

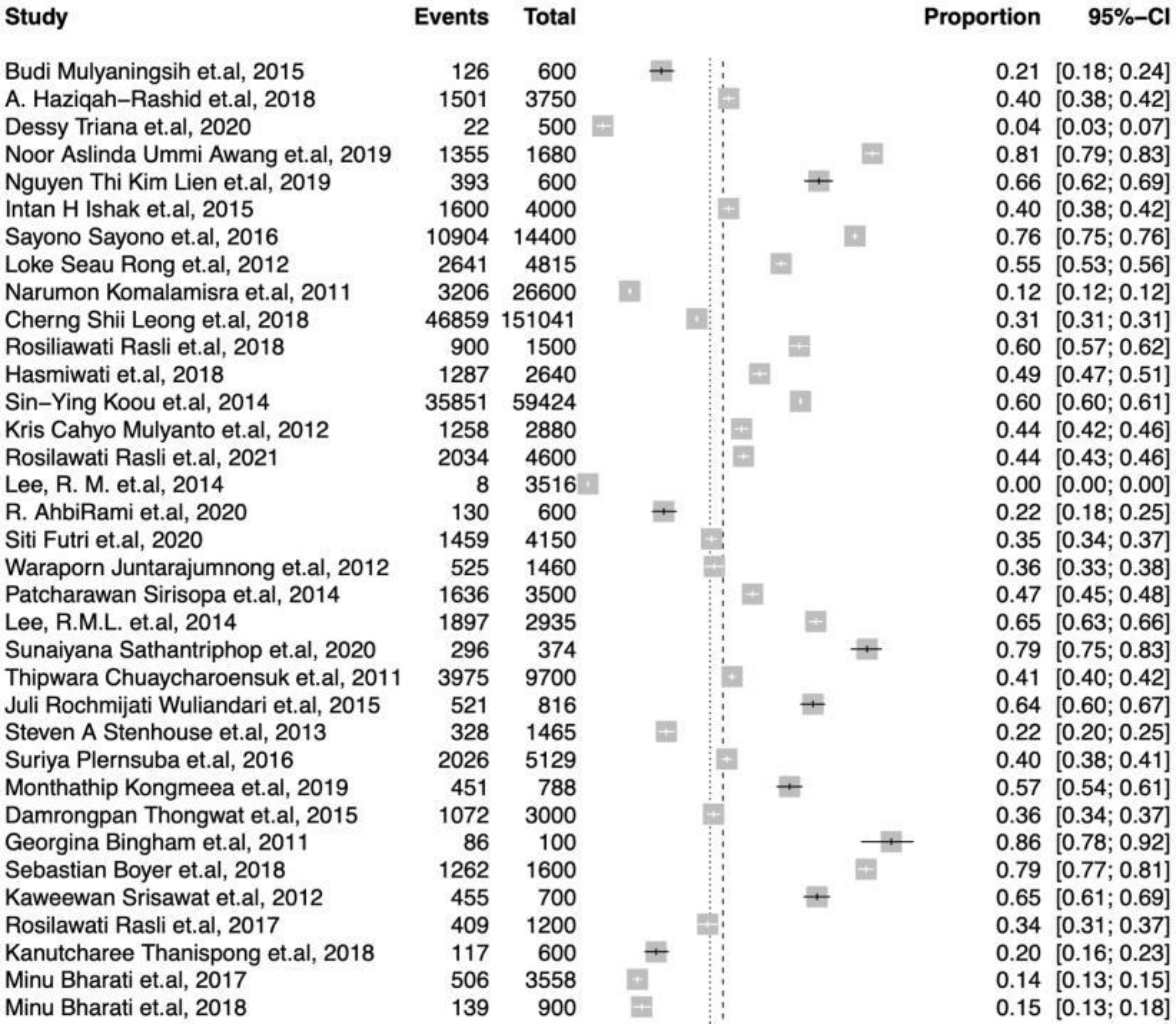

K. 2000-2010 (*Aedes albopictus*)

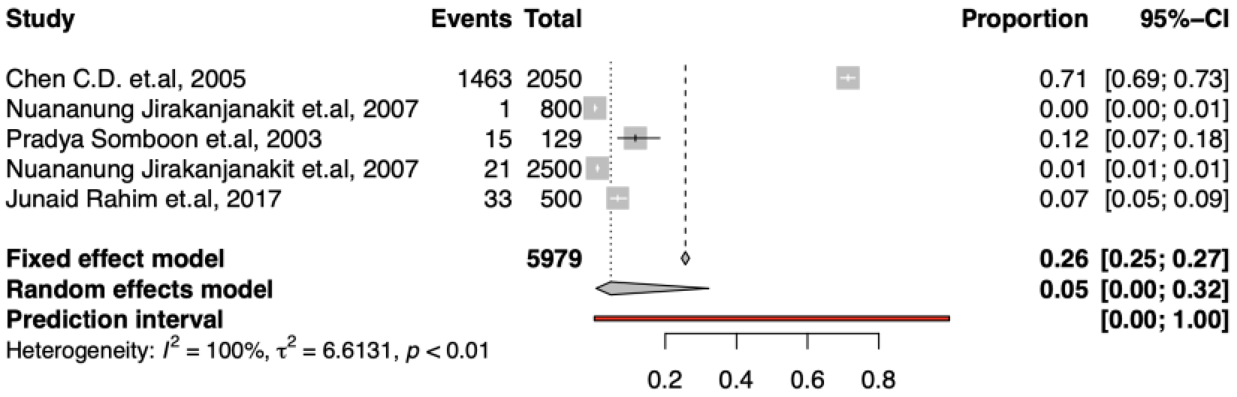

L. 2011-2021 (*Aedes albopictus*)

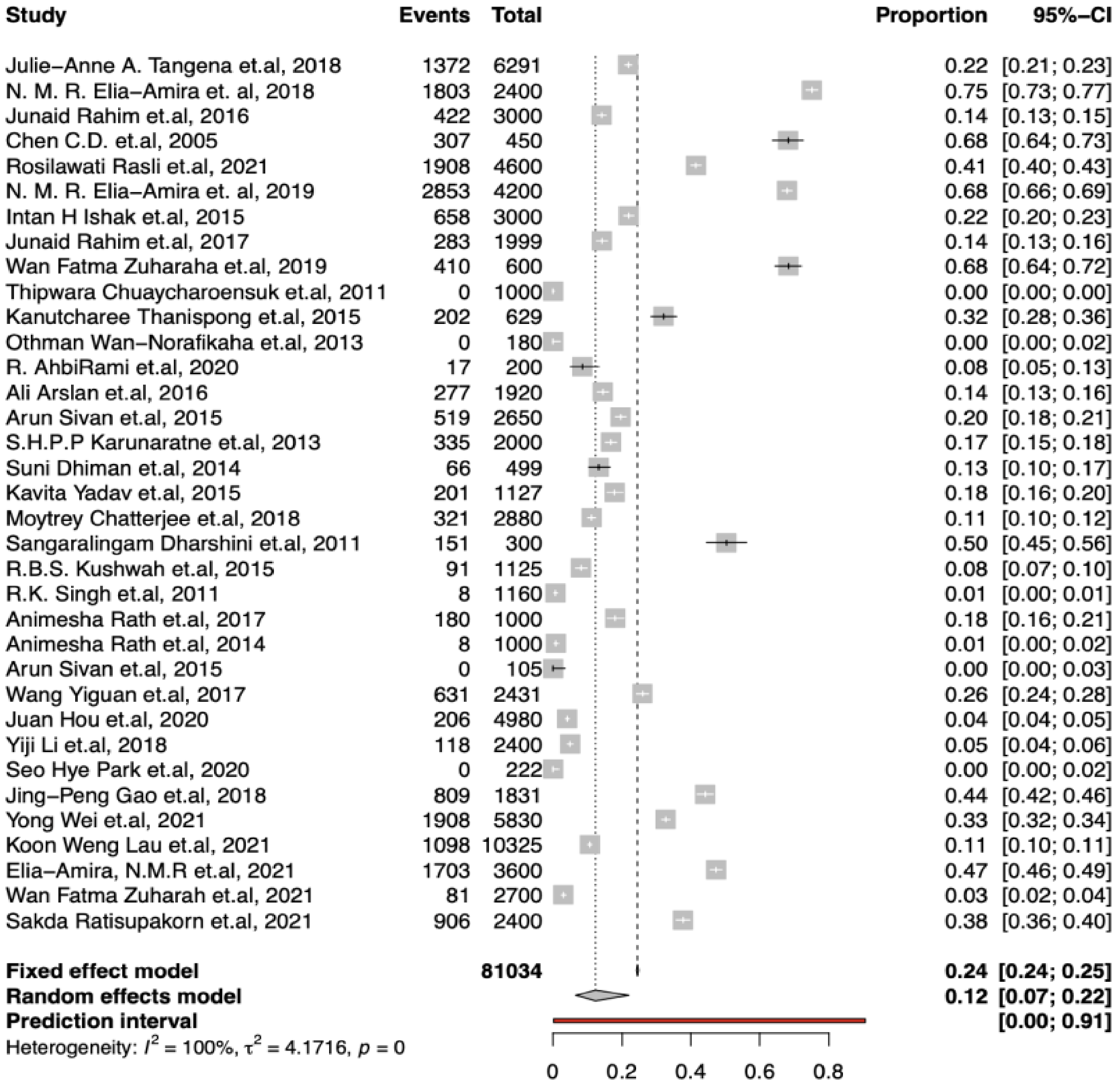

Figure S2. Forest Plots of meta-analysis. A. Malathion (Aedes aegypti); B. DDT (Aedes aegypti); C. Permethrin (Aedes aegypti); D. Deltamethrin (Aedes aegypti); E. Malathion (Aedes albopictus); F. DDT (Aedes albopictus); G. Permethrin (Aedes albopictus); H. Deltamethrin (Aedes albopictus); I. 2000-2010 (Aedes aegypti) Sub-group Analysis; J. 2011-2021 (Aedes aegypti); K. 2000-2010 (Aedes albopictus); L. 2011-2021 (Aedes albopictus).
