# Supplementary material for: Updating the Insecticide Resistance Status of Aedes aegypti and Aedes albopictus in Asia: A Systematic Review and Meta-Analysis
Source: Trop Med Infect Dis. 2022 Oct 17;7(10):306. doi: 10.3390/tropicalmed7100306 (PMC9607256; doi:10.3390/tropicalmed7100306)
Supplement: Supplementary file 1 [file tropicalmed-07-00306-s001.zip › Figure S3.pdf]

(a)

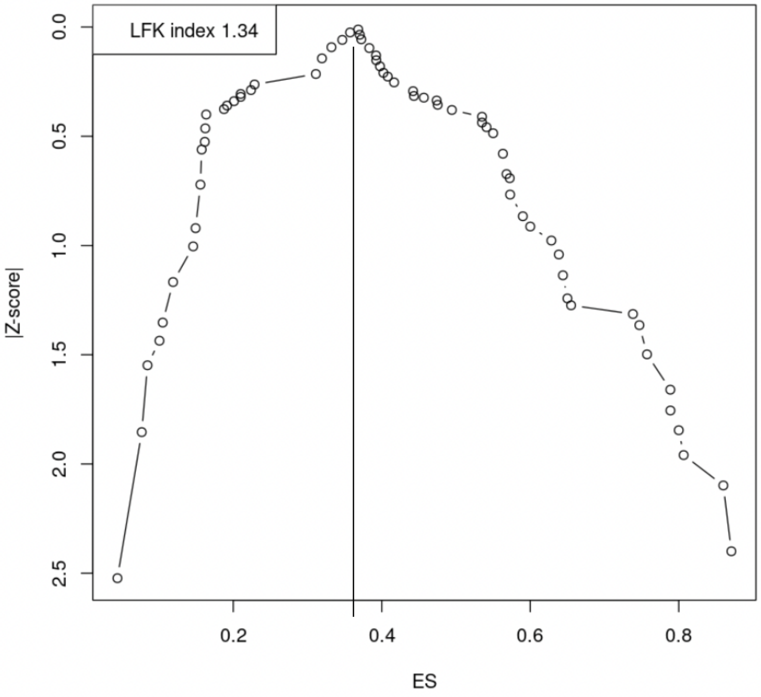
LFK index 1.34
|Z-score|
ES
0.0
0.5
1.0
1.5
2.0
2.5
0.2
0.4
0.6
0.8

(b)

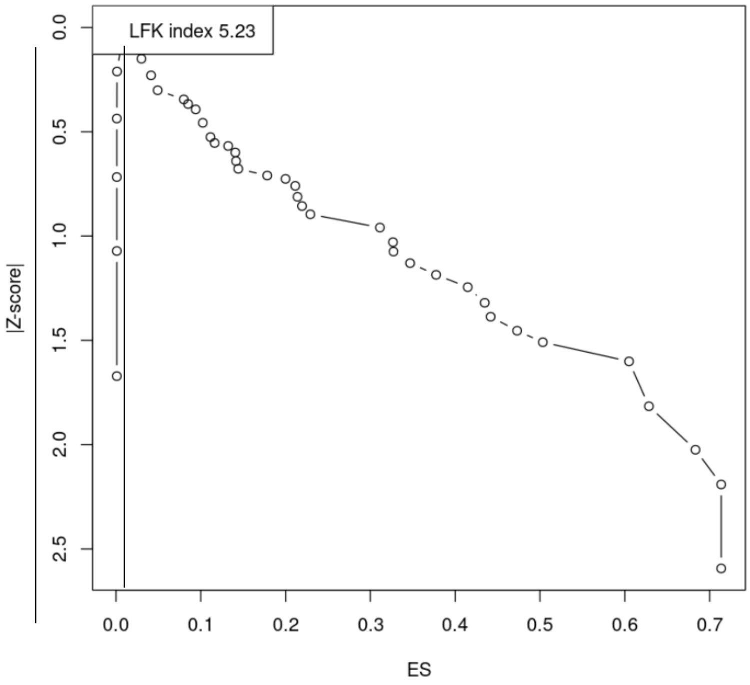
LFK index 5.23
|Z-score|
ES
0.0
0.5
1.0
1.5
2.0
2.5
0.0
0.1
0.2
0.3
0.4
0.5
0.6
0.7

Figure S3. Publication bias Doi plot and LFK index of (a) Ae. aegypti and (b) Ae. albopictus.
(ES = Estimation size).
