# Supplementary material for: Updating the Insecticide Resistance Status of Aedes aegypti and Aedes albopictus in Asia: A Systematic Review and Meta-Analysis
Source: Trop Med Infect Dis. 2022 Oct 17;7(10):306. doi: 10.3390/tropicalmed7100306 (PMC9607256; doi:10.3390/tropicalmed7100306)
Supplement: Supplementary file 1 [file tropicalmed-07-00306-s001.zip › Table S1.pdf]

Table S1. Searching string details.

Database	Searching string
PubMed	(((((Aedes aegypti) OR (Aedes albopictus)) AND (Organophosphate)) OR (Organochlorine)) OR (Pyrethroid)) AND (Resistance)
Scopus	(TITLE-ABS-KEY (Aedes AND aegypti) OR TITLE-ABS-KEY (Aedes AND albopictus) AND TITLE-ABS-KEY (organophosphate) OR TITLE-ABS-KEY (organochlorine) OR TITLE-ABS-KEY (pyrethroid) AND TITLE-ABS-KEY (resistance))
EbscoHost	"Aedes aegypti" OR "Aedes albopictus" AND "Organophosphate" OR "Organochlorine" OR "Pyrethroid" AND "Resistance"
Embase	(('aedes aegypti'/exp OR 'aedes aegypti' OR 'aedes albopictus':ti,ab,kw) AND organophosphate:ti,ab,kw OR 'organochlorine':ti,ab,kw OR pyrethroid:ti,ab,kw) AND resistance:ti,ab,kw)
