# Supplementary material for: Updating the Insecticide Resistance Status of Aedes aegypti and Aedes albopictus in Asia: A Systematic Review and Meta-Analysis
Source: Trop Med Infect Dis. 2022 Oct 17;7(10):306. doi: 10.3390/tropicalmed7100306 (PMC9607256; doi:10.3390/tropicalmed7100306)
Supplement: Supplementary file 1 [file tropicalmed-07-00306-s001.zip › Table S2.pdf]

Table S2. Characteristics of studies included in the systematic review.

No.	First Author	Insecticide type	Insecticide class	Study population	Bioassay method	Country
1*	Juli Rocmijati Wuliandari et.al, 2015	Permethrin, Deltamethrin,	Pyrethroid	*Ae. aegypti*	WHO	Indonesia
2	Budi Mulyaningsih et.al, 2018	Malathion, Cypermethrin	Organophosphate, Pyrethroid	*Ae. aegypti*	CDC	Indonesia
3	Steven A Stenhouse et.al, 2013	Deltamethrin	Pyrethroid	*Ae. aegypti*	WHO	Thailand
4*	Shavanthi Rajatileka et.al, 2008	DDT, Permethrin, Deltamethrin	Organochlorine, Pyrethroid	*Ae. aegypti*	WHO	Thailand
5	Suriya Plernsuba et.al, 2016	Deltamethrin	Pyrethroid	*Ae. aegypti*	WHO	Thailand
6	Sunaiyana Sathantriphop et.al, 2006	Fenitrothion, Malathion, Permethrin, Deltamethrin	Organophosphate, Pyrethroid	*Ae. aegypti*	WHO	Thailand
7	Waraporn Juntarajumnong et.al, 2012	Permethrin, Deltamethrin, Cypermethrin, alfa-cypermethrin, Bifenthrin, lamda-cyhalothrin	Pyrethroid	*Ae. aegypti*	WHO	Thailand

	Chutipong Sukkanon et.al, 2019	Transflutrin	Pyrethroid	*Ae. aegypti*	WHO	Thailand
9	Monthathip Kongmeea et.al, 2019	Deltamethrin	Pyrethroid	*Ae. aegypti*	WHO	Thailand
10	Kanutcharee Thanispong et.al, 2009	DDT, alfa-Cypermethrin	Organochlorine, Pyrethroid	*Ae. aegypti*	WHO	Thailand
11	A. Haziqah-Rashid et.al, 2018	Temephos, Fenitrothion, Malathion, Fenthion, Chlorpyrifos, Bromophos, Pirimiphos-methyl, DDT	Organophosphate, Organochlorine	*Ae. aegypti*	WHO	Indonesia
12	Dessy Triana et.al, 2020	Malathion, Cypermethrin	Organophosphate, Organochlorine	*Ae. aegypti*	CDC	Indonesia
13	Noor Aslinda Ummi Awang Besar et.al, 2019	Malathion, Pirimiphos-methyl, Deltamethrin	Organophosphate, Pyrethroid	*Ae. aegypti*	WHO	Malaysia
14	Patcharawan Sirisopa et.al, 2013	Permethrin, Deltamethrin, Cypermethrin, alfa-Cypermethrin, Bifenthrin, lamda-Cyhalothrin,	Pyrethroid	*Ae. aegypti*	WHO	Thailand
15	Siti-Futri, F.F. et.al, 2020	Cyfluthrin, Permethrin, Deltamethrin, lamda-Cyhalothrin	Pyrethroid	*Ae. aegypti*	WHO	Malaysia

16	Nguyen Thi Kim Lien et.al, 2019	Malathion, DDT, Permethrin, Deltamethrin, lamda-Cyhalothrin, Propoxur	Organophosphate, Organochlorine, Pyrethroid, Carbamate	*Ae. aegypti*	WHO	Vietnam
17	Intan H Ishak et.al, 2015	Malathion, DDT, Dieldrin, Permethrin, Deltamethrin, Bendiocarb	Organophosphate, Organochlorine, Pyrethroid, Carbamate	*Ae. aegypti, Ae. albopictus*	WHO	Malaysia
18	Lee, R.M.L. et.al, 2014	Pirimiphos-methyl, Cypermethrin	Organophosphate, Pyrethroid	*Ae. aegypti, Ae. albopictus*	WHO	Singapore
19	Pradya Somboon et.al, 2003	Fenitrothion, DDT, Permethrin, Deltamethrin, lamda-Cyhalothrin	Organophosphate, Organochlorine, Pyrethroid	*Ae. aegypti, Ae. albopictus*	WHO	Thailand
20*	Sayono, Syaono et.al, 2016	Malathion, Deltamethrin, alfa-Cypermethrin, lamda-Cyhalothrin	Organophosphate, Pyrethroid	*Ae. aegypti*	WHO	Indonesia
21	Damrongpan Thongwat et.al, 2015	Permethrin, Deltamethrin	Pyrethroid	*Ae. aegypti*	WHO	Thailand
22*	Sin-Ying Koou et.al, 2014	Pirimiphos-methyl, Permethrin, Cypermethrin, Etofenprox	Organophosphate, Pyrethroid	*Ae. aegypti*	WHO	Singapore
23	Sin-Ying Koou et.al, 2014	Temephos, Permethrin, Etofenprox	Organophosphate, Pyrethtoid	*Ae. aegypti*	WHO	Singapore

24	Kris Cahyo Mulyatno et.al, 2012	Fenitrothion	Organophosphate	*Ae. aegypti*	WHO	Indonesia
25	Loke Seau Rong et.al, 2012	Fenitrothion, Malathion, DDT, Permethrin, Deltmethrin	Organophosphate, DDT	*Ae. aegypti*	WHO	Malaysia
26*	Raweewan Srisawat et.al, 2012	Permethrin	Pyrethroid	*Ae. aegypti*	WHO	Thailand
27	Georgina Bingham et.al, 2011	Deltamethrin	Pyrethroid	*Ae. aegypti*	WHO	Vietnam
28	Narumon Komalamisra et.al, 2011	Tempehos	Organophosphate	*Ae. aegypti*	WHO	Thailand
29	Narumon Komalamisra	Temephos, Permethrin	Organochlorine, Pyrethroid	*Ae. aegypti*	WHO	Thailand
30	Othman Wan-Norafikah et.al, 2010	Permethrin	Pyrethroid	*Ae. aegypti*	WHO	Malaysia
31	Sunaiyana Sathantriphop et.al, 2020	Deltamethrin, Cypermethrin	Pyrethroid	*Ae. aegypti*	WHO	Thailand
32	Zheng Hua Amelia-Yap et.al, 2018	d-allethrin, metofluthrin, transfluthrin	Pyrethroid	*Ae. aegypti*	WHO	Indonesia
33	Kanutcharee Thanispong et.al, 2008	Malathion, DDT, Permethrin, Deltamethrin, Cypermethrin, Propoxur	Organophosphate, Organochlorine, Pyrethroid, Carbamate	*Ae. aegypti*	WHO	Thailand

34	Rosilawati Ra et.al, 2017	Permethrin	Pyrethroid	*Ae. aegypti*	WHO	Malaysia
35	Sébastien Boyer et.al, 2018	Permethrin, Deltamethrin	Pyrethroid	*Ae. aegypti*	WHO	Cambodia
36	Loke, S.R. et.al, 2010	Temephos	Organophosphate	*Ae. aegypti*	WHO	Malaysia
37	Karen A Polson et.al, 2001	Temephos	Organophosphate	*Ae. aegypti*	WHO	Cambodia
38*	Cherng Shii Leong et.al, 2018	Temephos, Malathion, DDT, Cyfluthrin, Permethrin, Deltamethrin, lamda-Cyhalothrin, Etofenprox, Propoxur	Organophosphate, Organochlorine, Pyrethroid, Carbamate	*Ae. aegypti*	WHO	Malaysia
39	Rosilawati Rasli et.al, 2018	Fenitrothion, Malathion, Cyfluthrin, Permethrin, Deltamethrin,	Organophosphate, Pyrethroid	*Ae. aegypti*	WHO	Malaysia
40	A. C. Chin et.al, 2017	d-allethrin, Metofluthrin, d-trans allethrin, Pralethrin	Pyrethroid	*Ae. aegypti*	WHO	Malaysia
41	Hasmiwati et.al, 2018	Temephos	Organophosphate	*Ae. aegypti*	WHO	Indonesia

42*	Cherng-Shii Leong et.al, 2019	Malathion, DDT, Cyfluthrin, Permethrin, Deltamethrin, lamda-Cyhalothrin, Etofenprox, Propoxur	Organophosphate, Organochlorine, Pyrethroid, Carbamate	*Ae. aegypti*	CDC	Malaysia
43*	Hasmiwati et.al, 2018	Temephos, Malathion, Permethrin, alfa-Cypermethrin,	Organophosphate, Pyrethroid	*Ae. aegypti*	WHO	Indonesia
44	Nuananong Jirakanjanakit et.al, 2007	Fenitrothion, Permethrin, Deltamethrin, Propoxur	Organophosphate, Pyrethroid, Carbamate	*Ae. aegypti*, *Ae. albopictus*	WHO	Thailand
45	Rosilawati Rasli et.al, 2021	Temephos	Organophosphate	*Ae. aegypti*, *Ae. albopictus*	WHO	Malaysia
46	R. AhbiRami et.al, 2020	Pirimiphos-methyl, Deltamethrin	Organophosphate, Pyrethroid	*Ae. aegypti*, *Ae. albopictus*	WHO	Malaysia
47	Chen, C.D. et.al, 2005	Temephos	Organophosphate	*Ae. aegypti*, *Ae. albopictus*	WHO	Malaysia
48	Wan Najdah Wan Mohamad Ali et.al, 2020	Malathion, DDT, Permethrin, lamda-Cyhalothrin, Propoxur	Organophosphate, Organochlorine, Pyrethroid, Carbamate	*Ae. aegypti*, *Ae. albopictus*	WHO	Malaysia
49	Vu Duc Huong et.al, 2004	Malathion, DDT, Permethrin, Deltamethrin, alfa-Cypermethrin, lamda-Cyhalothrin, Etofenprox	Organophosphate, Organochlorine, Pyrethroid	*Ae. aegypti*	WHO	Vietnam

50	Nuananong Jirakanjanakit et.al, 2007	Temephos	Organophosphate	*Ae. aegypti*, *Ae. albopictus*	WHO	Thailand
51	Chen, C.D. et.al, 2005	Temephos	Organophosphate	*Ae. aegypti*, *Ae. albopictus*	WHO	Malaysia
52*	Junaid Rahim et.al, 2017	Malathion, DDT, Permethrin, Deltamethrin	Organophosphate, Organochlorine, Pyrethroid	*Ae. albopictus*	WHO	Malaysia
53	N. M. R. Elia-Amira et.al, 2019	Fenitrothion, Malathion, DDT, Dieldrin, Cyfluthrin, Permethrin, Deltamethrin, lamda-Cyhalothrin, Etofenprox, Propoxur, Bendiocarb	Organophosphate, Organochlorine, Pyrethroid, Carbamate	*Ae. albopictus*	WHO	Malaysia
54	Chen, C.D. et.al, 2018	d-allethrin, Metofluthrin, d-trans allethrin, Pralethrin	Pyrethroid	*Ae. albopictus*	WHO	Malaysia
55*	Julie-Anne A. Tangena et.al, 2018	Temephos, Malathion, DDT, Permethrin, Deltamethrin,	Organophosphate, Organochlorine, Pyrethroid	*Ae. albopictus*	WHO	Laos

56	Kanutcharee Thanispong et.al, 2015	Permethrin, Deltamethrin, Cypermethrin, alfa-Cypermethrin, Bifenthrin	Pyrethroid	*Ae. albopictus*	WHO	Thailand
57	Low, V.L. et.al, 2015	Dieldrin	Organochlorine	*Ae. albopictus*	WHO	Malaysia
58	N. M. R. Elia-Amira et.al, 2018	Temephos, Fenitrothion, Malathion, Fenthion, Chlorpyrifos, Bromophos, Pirimiphos-methyl, DDT, Dieldrin	Organophosphate, Organochlorine	*Ae. albopictus*	WHO	Malaysia
59*	Juanid Rahim et.al, 2016	Temephos, Malathion	Organophosphate	*Ae. albopictus*	WHO	Malaysia
60	Wan Fatma Zuharaha et.al, 2019	Malathion, Permethrin	Organophosphate, Pyrethroid	*Ae. albopictus*	WHO	Malaysia
61	Othman Wan-Norafikaha	Permethrin	Pyrethroid	*Ae. albopictus*	WHO	Malaysia
62*	Raja Babu S. Kushwah et.al, 2020	DDT, Permethrin, Deltamethrin	Organochlorine, Pyrethroid	*Ae. aegypti*	WHO	India
63	Minu Bharati et.al, 2018	Temephos, Malathion, DDT, Permethrin, Deltamethrin, lamda-Cyhalothrin, Propoxur	Organophosphate, Organochlorine, Pyrethroid, Carbamate	*Ae. aegypti*	WHO	India
64*	Sachini D. Fernando et.al, 2018	Permethrin, Deltamethrin	Pyrethroid	*Ae. aegypti*	WHO	Sri Lanka

65	S.H.P.P. Karunaratne et.al, 2013	Malathion, DDT, Permethrin, Propoxur	Organophosphate, Organochlorine, Pyrethroid, Carbamate	*Ae. aegypti, Ae. albopictus*	WHO	Sri Lanka
66	Sunil Dhiman et.al, 2014	Malathion, DDT, Deltamethrin	Organophosphate, Organochlorine, Pyrethroid	*Ae. albopictus*	WHO	India
67	Animesha Rath et.al, 2017	DDT, Cyfluthrin	Organochlorine, Pyrethroid	*Ae. albopictus*	WHO	India
68	Kavita Yadav et.al, 2015	Malathion, DDT, Deltamethrin	Organophosphate, Organochlorine, Pyrethroid	*Ae. aegypti, Ae. albopictus*	WHO	India
69	Minu Bharati et.al, 2018	Temephos, Malathion, DDT, Permethrin, Deltamethrin, lamda-Cyhalothrin, Propoxur	Organophosphate, Organochlorine, Pyrethroid, Carbamate	*Ae. aegypti*	WHO	India
70	Moytrey Chatterjee et.al, 2018	Malathion, DDT, Deltamethrin	Organophosphate, Organochlorine, Pyrethroid	*Ae. albopictus*	WHO	India
71	Sangaralingam Dharshini et.al, 2011	Malathion, DDT, Permethrin	Organophosphate, Organochlorine, Pyrethroid	*Ae. aegypti, Ae. albopictus*	WHO	Sri Lanka

72*	Pabitra Saha et.al, 2019	Malathion, DDT, Deltamethrin, alfa-Cypermethrin	Organophosphate, Organochlorine, Pyrethroid	*Ae. aegypti*	WHO	India
73	Raja Babu S. et.al, 2015	DDT, Permethrin, Deltamethrin	Organochlorine, Pyrethroid	*Ae. aegypti*	WHO	India
74*	H. Sachini D. Fernando et.al, 2020	Malathion, Permethrin, Deltamethrin	Organophosphate, Pyrethroid	*Ae. aegypti*	WHO	Sri Lanka
75	Ali Arslan et.al, 2016	Temephos	Organophosphate	*Ae. aegypti, Ae. albopictus*	WHO	Pakistan
76*	R.B.S. Kushwah et.al, 2015	Malathion, Permethrin, Deltamthrin	Organophosphate, Pyrethroid	*Ae. albopictus*	WHO	India
77	R.K. Singh et.al, 2011	Malathion, DDT, Cyfluthrin, Permethrin, Deltamethrin, lamda-Cyhalothrin	Organophosphate, Organochlorine, Pyrethroid	*Ae. aegypti, Ae. albopictus*	WHO	India
78	Arun Sivan et.al, 2015	Temephos, Fenitrothion, Malathion, Fenthion, DDT, Dieldrin, Cyfluthrin, Permethrin, Deltamethrin, lamda-Cyhalothrin, Propoxur, Bendiocarb	Organophosphate, Organochlorine, Pyrethroid, Carbamate	*Ae. aegypti, Ae. albopictus*	WHO	India

79	Rakesh Katyal et.al, 2001	Temephos, Fenitrothion, Malathion, Fenthion, DDT, Dieldrin, Permethrin, Deltamethrin, lamda-Cyhalothrin, propoxur	Organophosphate, Organochlorine, Pyrethroid, Carbamate	*Ae. aegypti*	WHO	India
80	Wang Yiguan et.al, 2017	Temephos, Diclorvos, DDT, Permethrin, Deltamethrin, Cypermethrin, beta-Cypermethrin, Propoxur	Organophosphate, organochlorine, Pyrethroid, Carbamate	*Ae. albopictus*	WHO	China
81	Jing-Peng Gao et.al, 2018	Permethrin, Deltamethrin, beta-Cypermethrin, lamda-Cyhalothrin	Pyrethroid	*Ae. albopictus*	WHO	China
82	Yiji Li et.al, 2018	Malathion, DDT, Deltamethrin, Propoxur	Organophosphate, Organochlorine, Pyrethroid, Carbamate	*Ae. albopictus*	WHO	China
83	Juan Hou et.al, 2020	Fenitrothion, Malathion, Permethrin, Deltamethrin, alfa-Cypermethrin, beta-Cypermethrin, lamda-Cyhalothrin, Propoxur	Organophosphate, Pyrethroid, Carbamate	*Ae. albopictus*	WHO	China
84	Seo Hye Park et.al, 2020	Chlorpyrifos, Deltamethrin	Organophosphate, Pyrethroid	*Ae. albopictus*	WHO	Korea
85	Koon Weng Lau et.al, 2021	Fenitrothion, Malathion, DDT, Dieldrin, Cyfluthrin, Permethrin, Deltamethrin, lamda-	Organophosphate, Organochlorine, Pyrethroid, Carbamate	*Ae. albopictus*	WHO	Malaysia

		Cyhalothrin, Etofenprox, Propoxur, Bendiocarb				
86	Sakda Ratisupakorn et.al, 2021	Chlorpyrifos, lamda-Cyhalotrhin, Carbaryl, Imidaclorpid	Organophosphate, Pyrethroid, Neonicotinoid	*Ae. albopictus*	WHO	Thailand
87	Elia-Amira, N.M.R et.al, 2021	Temephos, Fenitrothion, Malathion, Fenthion, Chlorpyrifos, Bromophos, DDT, Dieldrin	Organophosphate, Organochlorine	*Ae. albopictus*	WHO	Malaysia
88	Wan Fatma Zuharah et.al, 2021	Malathion, Permethrin, Delamethrin,	Organophosphate, Pyrethroid	*Ae. albopictus*	WHO	Malaysia
89	Wan Fatma Zuharah et.al, 2021	Permethrin, Deltamethrin	Pyrethroid	*Ae. aegypti*	WHO	Malaysia
90	Yong Wei et.al, 2021	Deltamethrin	Pyrethroid	*Ae. albopictus*	WHO	China
91*	Yuyan Wu et.al, 2021	N/A	N/A	*Ae. albopictus*	N/A	China
92*	Han-Hsuan Chung et.al, 2019	N/A	N/A	*Ae. aegypti*	N/A	Taiwan
93*	Xiaodong Yang et.al, 2021	N/A	N/A	*Ae. albopictus*	N/A	China
94*	H. Kawada et.al, 2009	N/A	N/A	*Ae. aegypti*	N/A	Vietnam

*= Indicate the study identify the kdr mutation sites
