# Supplementary material for: Updating the Insecticide Resistance Status of Aedes aegypti and Aedes albopictus in Asia: A Systematic Review and Meta-Analysis
Source: Trop Med Infect Dis. 2022 Oct 17;7(10):306. doi: 10.3390/tropicalmed7100306 (PMC9607256; doi:10.3390/tropicalmed7100306)
Supplement: Supplementary file 1 [file tropicalmed-07-00306-s001.zip › Table S3.pdf]

Table S3. Risk of Bias (RoB) assessment. Q1: Was the study population a close representative of the national population? Q2: Was the sampling frame a true or close representation of target population? Q3: Was some form of random selection used to select the sample? Q4: was the likelihood of non-response bias minimal? Q5: Were data collected directly from the subjects? Q6: was an acceptable case definition used in the study? Q7: Was the condition measured in a standard, reliable way for all participants? Q8: Was the same mode of data collection used for all subjects? Q9: Was the length of the prevalence period for the parameter of interest appropriate? Q10: Were the numerator(s) and denominator(s) for the parameter of interest appropriate?

	**Risk of Bias Assessment**													
**No.**	**Title**	**Authors**	**Q1**	**Q2**	**Q3**	**Q4**	**Q5**	**Q6**	**Q7**	**Q8**	**Q9**	**Q10**	**Scor e**	**Result**
1*	Association between Three Mutations, F1565C, V1023G and S996P, in the Voltage-Sensitive Sodium Channel Gene and Knockdown Resistance in *Aedes aegypti* from Yogyakarta, Indonesia	Juli Rochmijati Wuliandari et.al, 2015	No	Yes	Yes	Yes	Yes	Yes	Yes	Yes	Yes	Yes	9	Low Risk
2	Detection of Polymorphism on Voltage-gated Sodium Channel Gene of Indonesian *Aedes aegypti* Associated with Resistance to Pyrethroids	Budi Mulyaningsih et.al, 2018	No	Yes	Yes	Yes	Yes	Yes	Yes	Yes	Yes	Yes	9	Low Risk
3	Detection of the V1016G mutation in the voltage-gated sodium channel gene of Aedes aegypti (Diptera: Culicidae) by allele-specific PCR assay, and its distribution and effect<br>on deltamethrin resistance in Thailand	Steven A Stenhouse et.al, 2013	Yes	Yes	Yes	Yes	Yes	Yes	Yes	Yes	Yes	Yes	10	Low Risk
4	Development and application of a simple colorimetric assay reveals widespread distribution of sodium channel mutations in Thai populations of Aedes aegypti	Shavanthi Rajatilekaa et.al, 2008	Yes	Yes	Yes	Yes	Yes	Yes	Yes	Yes	Yes	Yes	10	Low Risk
5	Temporal frequency of knockdown resistance mutations, F1534C and V1016G, in Aedes aegypti in Chiang Mai city, Thailand and the impact of the mutations on	Suriya Plernsuba et.al, 2016	No	Yes	Yes	Yes	Yes	Yes	Yes	Yes	Yes	Yes	9	Low Risk

	the efficiency of thermal fogging spray with pyrethroids												
6	Detection of insecticides resistance status in Culex quinquefasciatus and Aedes aegypti to four major groups of insecticides	Sunaiyana Sathantriphop et.al, 2006	No	Yes	Yes	Yes	Yes	Yes	Yes	Yes	Ma ybe	Yes	8	Moder ate Risk
7	Discriminating Lethal Concentrations and Efficacy of Six Pyrethroids for Control of Aedes aegypti in Thailand	Waraporn Juntarajumnong et.al, 2012	No	Yes	Yes	Yes	Yes	Yes	Yes	Yes	Yes	Yes	9	Low Risk
8	DISCRIMINATING LETHAL CONCENTRATIONS FOR TRANSFLUTHRIN, A VOLATILE PYRETHROID COMPOUND FOR MOSQUITO CONTROL IN THAILAND	CHUTIPONG SUKKANON et.al, 2019	No	Yes	Yes	Yes	Yes	Yes	Yes	Yes	Yes	Yes	9	Low Risk
9	Enhanced mortality in deltamethrin-resistant Aedes aegypti in Thailand using T a piperonyl butoxide synergist	Monthathip Kongmeea et.al, 2019	Yes	Yes	Yes	Yes	Yes	Yes	Yes	Yes	Yes	No	8	Moder ate Risk
10	Irritancy and Repellency Behavioral Responses of Three Strains of Aedes aegypti Exposed to DDT and α-Cypermethrin	Kanutcharee Thanispong et.al, 2009	No	Yes	Yes	Yes	Yes	Yes	Yes	Yes	Ma ybe	Yes	9	Low Risk
11	Monitoring Insecticide Resistance Profiles of Aedes aegypti (Diptera: Culicidae) in the Sunda Islands of Indonesia Based on Diagnostic Doses of Larvicides	A. Haziqah-Rashid et.al, 2018	Yes	Yes	Yes	Yes	Yes	Yes	Yes	Yes	Yes	No	8	Moder ate Risk
12	RESISTANCE STATUS OF AEDES AEGYPTI TO MALATHION AND CYPERMETHRIN IN BENGKULU CITY, INDONESIA	Dessy Triana et.al, 2020	No	Yes	Yes	Yes	Yes	Yes	Yes	Yes	Yes	Yes	9	Low Risk
13	RESISTANCE STATUS OF Aedes aegypti TOWARDS DIFFERENT INSECTICIDES IN SELECTED DENGUE OUTBREAK AREA IN PETALING DISTRICT (DIPTERA: CULICINAE)	Noor Aslinda Ummi Awang Besar et.al, 2019	No	Yes	Yes	Yes	Yes	Yes	Yes	Yes	Yes	Yes	9	Low Risk

14	Resistance to Synthetic Pyrethroids in *Aedes aegypti* (Diptera: Culicidae) in Thailand	Patcharawan Sirisopa et.al, 2014	Yes	Yes	Yes	Yes	Yes	Yes	Yes	Yes	Yes	No	9	Low Risk
15	Status of pyrethroid resistance in Aedes (Stegomyia) aegypti (Linneaus) from dengue hotspots in Klang Valley, Malaysia	Siti-Futri et.al, 2020	No	Yes	Yes	Yes	Yes	Yes	Yes	Yes	Yes	No	8	Moderate Risk
16	Transcriptome Sequencing and Analysis of Changes Associated with Insecticide Resistance in the Dengue Mosquito (Aedes aegypti) in Vietnam	Nguyen Thi Kim Lien et.al, 2019	No	Yes	Yes	Yes	Yes	Yes	Yes	Yes	Yes	No	8	Moderate Risk
17	Contrasting patterns of insecticide resistance and knockdown resistance (kdr) in the dengue vectors Aedes aegypti and Aedes albopictus from Malaysia	Intan H Ishak et.al, 2015	Maybe	Yes	Yes	Yes	Yes	Yes	Yes	Yes	Yes	No	8	Moderate Risk
18	Bioassay and biochemical studies of the status of pirimiphos-methyl and cypermethrin resistance in Aedes (Stegomyia) aegypti and Aedes (Stegomyia) albopictus (Diptera: Culicidae) in Singapore	Lee, R.M.L. et.al, 2014	Yes	Yes	Yes	Yes	Yes	Yes	Yes	Yes	Yes	Yes	10	Low Risk
19	Insecticide susceptibility tests of Anopheles minimus s.l., Aedes aegypti, Aedes albopictus, and Culex quinquefasciatus in northern Thailand	Pradya Somboon et.al, 2003	No	Yes	Yes	Yes	Yes	Yes	Yes	Yes	Yes	Yes	9	Low Risk
20	Distribution of Voltage-Gated Sodium Channel (Nav) Alleles among the Aedes aegypti Populations In Central Java Province and Its Association with Resistance to Pyrethroid Insecticides	Sayono Sayono et.al, 2016	No	Yes	Yes	Yes	Yes	Yes	Yes	Yes	Yes	No	8	Moderate Risk
21	Susceptibility to temephos, permethrin and deltamethrin of Aedes aegypti (Diptera: Culicidae) from Muang district, Phitsanulok Province, Thailand	Damrongpan Thongwat et.al, 2015	No	Yes	Yes	Yes	Yes	Yes	Yes	Yes	Yes	No	8	Moderate Risk
22	Insecticide resistance and its underlying mechanisms in field populations of Aedes	Sin-Ying Koou et.al, 2014	Yes	Yes	Yes	Yes	Yes	Yes	Yes	Yes	Yes	Yes	10	Low Risk

	aegypti adults (Diptera: Culicidae) in Singapore												
23	Pyrethroid Resistance in *Aedes aegypti* Larvae (Diptera: Culicidae) From Singapore	SIN-YING KOOU et.al, 2014 (a)	Yes	Yes	Yes	Yes	Yes	Yes	Yes	Yes	Yes	Yes	10	Low Risk
24	RESISTANCE OF AEDES AEGYPTI (L.) LARVAE TO TEMEPHOS IN SURABAYA, INDONESIA	Kris Cahyo Mulyatno et.al, 2012	No	No	Yes	Yes	Yes	Yes	Yes	Yes	Yes	No	7	Moderate Risk
25	Insecticide susceptibility status of field-collected Aedes (Stegomyia) aegypti (L.) at a dengue endemic site in Shah Alam, Selangor, Malaysia	Loke Seau Rong et.al, 2012	No	Yes	Yes	Yes	Yes	Yes	Yes	Yes	Yes	No	8	Moderate Risk
26	FIELD-COLLECTED PERMETHRIN RESISTANT AEDES AEGYPTI FROM CENTRAL THAILAND CONTAIN POINT MUTATIONS IN THE DOMAIN IIS6 OF THE SODIUM CHANNEL GENE (KDR)	Raweewan Srisawat et.al, 2012	No	No	Yes	Yes	Yes	Yes	Yes	Yes	Yes	Yes	8	Moderate Risk
27	Can piperonyl butoxide enhance the efficacy of pyrethroids against pyrethroid-resistant Aedes aegypti?	Georgina Bingham et.al, 2011	No	N/A	Yes	Yes	Yes	Yes	Yes	Yes	Maybe	No	7	Moderate Risk
28	Frequency of pyrethroid resistance in Aedes aegypti and Aedes albopictus (Diptera: Culicidae) in Thailand	Thipwara Chuaycharoensuk et.al, 2011	Yes	N/A	Yes	Yes	Yes	Yes	Yes	Yes	Yes	No	9	Low Risk
29	Insecticide susceptibility of the dengue vector, Aedes aegypti (L.) in Metropolitan Bangkok	Narumon Komalamisra et.al, 2011	No	Yes	Yes	Yes	Yes	Yes	Yes	Yes	Yes	No	8	Moderate Risk
30	Permethrin resistance inAedes aegypti(Linnaeus) collected from KualaLumpur, Malaysia	Othman Wan-Norafikah et.al, 2010	No	Yes	Yes	Yes	Yes	Yes	Yes	Yes	Yes	No	8	Moderate Risk
31	Efficacy of Mosquito Coils: Cross-resistance to Pyrethroids in Aedes aegypti (Diptera: Culicidae) From Indonesia	Zheng Hua Amelia-Yap et.al, 2018	Yes	Yes	Yes	Yes	Yes	Yes	Yes	Yes	Yes	No	9	Low Risk
32	Insecticide resistance of *Aedes aegypti* and *Culex quinquifasciatus* in Thailand	Kanutcharee	No	Yes	Yes	Yes	Yes	Yes	Yes	Yes	Yes	No	8	Moderate Risk

		THANISPONG et.al, 2008											
33	Pyrethroid Resistance Status of Aedes (Stegomyia) aegypti (Linneaus) from Dengue Endemic Areas in Peninsular Malaysia	Rosilawati Ra et.al, 2017	No	Yes	Yes	Yes	Yes	Yes	Yes	Yes	Yes	No	8	Moderate Risk
34	Resistance of Aedes aegypti (Diptera: Culicidae) Populations to Deltamethrin, Permethrin, and Temephos in Cambodia	Sébastien Boyer et.al, 2018	No	N/A	Yes	Yes	Yes	Yes	Yes	Yes	Yes	No	8	Moderate Risk
35	Susceptibility of field-collected *Aedes aegypti* (L.) (Diptera: Culicidae) to *Bacillus thuringiensis israelensis* and temephos	Loke, S.R. et.al, 2010	No	Yes	Yes	Yes	Yes	Yes	Yes	Yes	Yes	No	8	Moderate Risk
36	Susceptibility of Two Cambodian Populations of Aedes aegypti Mosquito Larvae to Temephos During 2001	Karen A Polson et.al, 2001	No	Yes	Yes	Yes	Yes	Yes	Yes	Yes	Yes	Yes	9	Low Risk
37	Aedes aegypti(Linnaeus) larvae from dengue outbreak areas in Selangor showing resistance to pyrethroids but susceptible to organophosphates	Cherng Shii Leong et.al, 2018	No	Yes	Yes	Yes	Yes	Yes	Yes	Yes	Yes	Yes	9	Low Risk
38	Susceptibility Status and Resistance Mechanisms in Permethrin-Selected, Laboratory Susceptible and Field-Collected Aedes aegypti from Malaysia	Rosilawati Rasli et.al, 2018	No	Yes	Yes	Yes	Yes	Yes	Yes	Yes	Yes	No	8	Moderate Risk
39	Comparative Efficacy of Commercial Mosquito Coils Against Aedes aegypti (Diptera: Culicidae) in Malaysia: A Nationwide Report	A. C. Chin et.al, 2017	Yes	Yes	Yes	Yes	Yes	Yes	Yes	Yes	Yes	No	9	Low Risk
40	Detection of Ace-1 gene with insecticides resistance in Aedes aegypti populations from DHF-endemic areas in Padang, Indonesia	HASMIWATI et.al, 2018	No	Yes	Yes	Yes	Yes	Yes	Yes	Yes	Yes	No	8	Moderate Risk

41	Enzymatic and molecular characterization of insecticide resistance mechanisms in field populations of Aedes aegypti from Selangor, Malaysia	Cherng-Shii Leong et.al, 2019	No	Yes	Yes	Yes	Yes	Yes	Yes	Yes	Yes	No	8	Moderate Risk
42	Short Communication: Genotyping of kdr allele in insecticide resistant-Aedes aegypti populations from West Sumatra, Indonesia	HASMIWATI et.al, 2018 (a)	No	Yes	Yes	Yes	Yes	Yes	Yes	Yes	Yes	No	8	Moderate Risk
43	Insecticide Susceptible/Resistance Status in Aedes (Stegomyia) aegypti and Aedes (Stegomyia) albopictus (Diptera: Culicidae) in Thailand During 2003–2005	NUANANONG JIRAKANJANAKIT et.al, 2007	Yes	Yes	Yes	Yes	Yes	Yes	Yes	Yes	Yes	No	9	Low Risk
44	Insecticide resistance in dengue vectors from hotspots in Selangor, Malaysia	Rosilawati RasliID et.al, 2021	No	Yes	Yes	Yes	Yes	Yes	Yes	Yes	Yes	No	8	Moderate Risk
45	Knockdown resistance (kdr) in dengue vectors, Aedes aegypti and Aedes albopictus: A post-flood risk assessment	R. AhbiRami et.al, 2020	No	Yes	Yes	Yes	Yes	Yes	Yes	Yes	Yes	No	8	Moderate Risk
46	Susceptibility of Aedes aegypti and Aedes albopictus to temephos in four study sites in Kuala Lumpur City Center and Selangor State, Malaysia	Chen, C.D et.al, 2005	No	Yes	Yes	Yes	Yes	Yes	Yes	Yes	Yes	No	8	Moderate Risk
47	SPATIAL DISTRIBUTION, ENZYMATIC ACTIVITY, AND INSECTICIDE RESISTANCE STATUS OF Aedes aegypti AND Aedes albopictus FROM DENGUE HOTSPOT AREAS IN KUALA LUMPUR AND SELANGOR, MALAYSIA	Wan Najdah Wan Mohamad Ali et.al, 2020	Yes	Yes	Yes	Yes	Yes	Yes	Yes	Yes	Yes	Yes	10	Low Risk
48	Susceptibility of Aedes aegypti to Insecticides in Viet Nam	Vu Duc Huong et.al, 2004	Yes	Yes	Yes	Yes	Yes	Yes	Yes	Yes	Yes	Yes	10	Low Risk
49	Trend of Temephos Resistance in Aedes (Stegomyia) Mosquitoes in Thailand During 2003–2005	NUANANONG JIRAKANJANAKIT et.al, 2007	Yes	Yes	Yes	Yes	Yes	Yes	Yes	Yes	Yes	No	9	Low Risk

50	Weekly variation on susceptibility status of Aedes mosquitoes against temephos in Selangor, Malaysia	Chen, C.D et.al, 2005 (a)	No	Yes	Yes	Yes	Yes	Yes	Yes	Yes	Yes	No	8	Moderate Risk
51	Adulticidal Susceptibility Evaluation of Aedes albopictus Using New Diagnostic Doses in Penang Island, Malaysia	Junaid Rahim et.al, 2017	No	Yes	Yes	Yes	Yes	Yes	Yes	Yes	Yes	Yes	9	Low Risk
52	Adulticide Resistance Status of Aedes albopictus (Diptera: Culicidae) in Sabah, Malaysia: A Statewide Assessment	N. M. R. Elia-Amira et.al, 2019	No	Yes	Yes	Yes	Yes	Yes	Yes	Yes	Yes	No	8	Moderate Risk
53	Bioefficacy Evaluation of Commercial Mosquito Coils Containing Metofluthrin, d-Allethrin, d-Trans Allethrin, and Prallethrin Against *Aedes albopictus* (Diptera: Culicidae) in Malaysia	C. D. Chen et.al, 2018	Yes	Yes	Yes	Yes	Yes	Yes	Yes	Yes	Yes	No	9	Low Risk
54	Bionomics and insecticide resistance of the arboviral vector Aedes albopictus in northern Lao PDR	Julie-Anne A. Tangena et.al, 2018	No	Yes	Yes	Yes	Yes	Yes	Yes	Yes	Yes	Yes	9	Low Risk
55	ESTABLISHMENT OF DIAGNOSTIC DOSES OF FIVE PYRETHROIDS FOR MONITORING PHYSIOLOGICAL RESISTANCE IN AEDES ALBOPICTUS IN THAILAND	KANUTCHAREE THANISPONG et.al, 2015	No	Yes	Yes	Yes	Yes	Yes	Yes	Yes	Yes	Yes	9	Low Risk
56	First molecular genotyping of A302S mutation in the gamma aminobutyric acid (GABA) receptor in Aedes albopictus from Malaysia	Low, V.L. et.al, 2015	No	Yes	Yes	Yes	Yes	Yes	Yes	Yes	Yes	No	8	Moderate Risk
57	Organophosphate and Organochlorine Resistance in Larval Stage of Aedes albopictus (Diptera: Culicidae) in Sabah, Malaysia	N. M. R. Elia-Amira et.al, 2018	Yes	Yes	Yes	Yes	Yes	Yes	Yes	Yes	Maybe	No	8	Moderate Risk
58	Revised Discriminating Lethal Doses For Resistance Monitoring Program on Aedes	Junaid Rahim et.al, 2016	No	Yes	Yes	Yes	Yes	Yes	Yes	Yes	Yes	Yes	9	Low Risk

	albopictus Against Temephos and Malathion in Penang Island, Malaysia													
59	Susceptibility and fitness cost of Aedes albopictus on their survivability after T the exposure to the insecticide	Wan Fatma Zuharah et.al, 2019	No	Yes	Yes	Yes	Yes	Yes	Yes	Yes	Yes	Yes	9	Low Risk
60	Susceptibility of Aedes albopictus Skuse (Diptera: Culicidae) to permethrin in Kuala Lumpur, Malaysia	Othman Wan-Norafikah et.al, 2013	No	Yes	Yes	Yes	Yes	Yes	Yes	Yes	Yes	No	8	Moderate Risk
61	A new knockdown resistance (*kdr*) mutation, F1534L, in the voltage-gated sodium channel of *Aedes aegypti*, co-occurring with F1534C, S989P and V1016G	Raja Babu S. Kushwah et.al, 2020	No	Yes	Yes	Yes	Yes	Yes	Yes	Yes	Yes	Yes	9	Low Risk
62	Assessment of insecticide resistance in primary dengue vector, Aedes aegypti T (Linn.) from Northern Districts of West Bengal, India	Minu Bharati et.al, 2018	No	Yes	Yes	Yes	Yes	Yes	Yes	Yes	Yes	No	8	Moderate Risk
63	First report of V1016G and S989P knockdown resistant (kdr) mutations in pyrethroid-resistant Sri Lankan Aedes aegypti mosquitoes	Sachini D. Fernando1 et.al, 2018	No	Yes	Yes	Yes	Yes	Yes	Yes	Yes	Yes	Yes	9	Low Risk
64	Insecticide resistance and, efficacy of space spraying and larviciding in the control of dengue vectors Aedes aegypti and Aedes albopictus in Sri Lanka	S.H.P.P. Karunaratne et.al, 2013	No	Yes	Yes	Yes	Yes	Yes	Yes	Yes	Yes	No	8	Moderate Risk
65	Insecticide susceptibility and dengue vector status of wild Stegomyia albopicta in a strategically important area of Assam, India	Sunil Dhiman et.al, 2014	No	Yes	Yes	Yes	Yes	Yes	Yes	Yes	Yes	No	8	Moderate Risk
66	Insecticide susceptibility status of invasive Aedes albopictus across dengue endemic districts of Odisha, India	Animesha Rath et.al, 2017	No	Yes	Yes	Yes	Yes	Yes	Yes	Yes	Yes	No	8	Moderate Risk
67	Multi-insecticide susceptibility evaluation of dengue vectors Stegomyia albopicta and St. aegypti in Assam, India	Kavita Yadav et.al, 2015	No	Yes	Yes	Yes	Yes	Yes	Yes	Yes	Yes	Yes	9	Low Risk

68	Multiple insecticide resistance mechanisms in primary dengue vector, Aedes aegypti (Linn.) from dengue endemic districts of sub- Himalayan West Bengal, India	Minu Bharati et.al, 2018 (a)	No	Yes	Yes	Yes	Yes	Yes	Yes	Yes	Yes	No	8	Moderate Risk
69	Polymorphisms in voltage-gated sodium channel gene and susceptibility of Aedes albopictus to insecticides in three districts of northern West Bengal, India	Moytrey Chatterjee et.al, 2018	No	Yes	Yes	Yes	Yes	Yes	Yes	Yes	Yes	Yes	9	Low Risk
70	Prevalence and insecticide susceptibility of dengue vectors in the district of Batticaloa in eastern Sri Lanka	Sangaralingam Dharshini et.al, 2011	No	Yes	Yes	Yes	Yes	Yes	Yes	Yes	Yes	Yes	9	Low Risk
71	Prevalence of kdr mutations and insecticide susceptibility among natural population of Aedes aegypti in West Bengal	Pabitra Saha et.al, 2019	No	Yes	Yes	Yes	Yes	Yes	Yes	Yes	Yes	Yes	9	Low Risk
72	Pyrethroid-Resistance and Presence of Two Knockdown Resistance (kdr) Mutations, F1534C and a Novel Mutation T1520I, in Indian Aedes aegypti	Raja Babu S. Kushwah et.al, 2015	No	Yes	Yes	Yes	Yes	Yes	Yes	Yes	Yes	Yes	9	Low Risk
73	Resistance to commonly used insecticides and underlying mechanisms of resistance in Aedes aegypti (L.) from Sri Lanka	H. Sachini D. Fernando et.al, 2020	No	Yes	Yes	Yes	Yes	Yes	Yes	Yes	Yes	Yes	9	Low Risk
74	Spatial distribution and insecticide susceptibility status of Aedes aegypti and Aedes albopictus in dengue affected urban areas of Rawalpindi, Pakistan	Ali Arslan et.al, 2016	No	Yes	Yes	Yes	Yes	Yes	Yes	Yes	Yes	Yes	9	Low Risk
75	Status of DDT and pyrethroid resistance in Indian Aedes albopictus and absence of knockdown resistance (kdr) mutation	R.B.S. Kushwah et.al, 2015	No	Yes	Yes	Yes	Yes	Yes	Yes	Yes	Yes	Yes	9	Low Risk
76	Susceptibility status of dengue vectors against various insecticides in Koderma (Jharkhand), India	R.K. Singh et.al, 2011	No	No	Yes	Yes	Yes	Yes	Yes	Yes	Yes	Yes	8	Moderate Risk

77	Studies on insecticide susceptibility of Aedes aegypti (Linn) and Aedes albopictus (Skuse) vectors of dengue and chikungunya in Andaman and Nicobar Islands, India	Arun Sivan et.al, 2015	No	Yes	Yes	Yes	Yes	Yes	Yes	Yes	Yes	Yes	9	Low Risk
78	Susceptibility Status of Immature and Adult Stages of Aedes aegypti Against Conventional Insecticides in Delhi, India	Rakesh Katyal et.al, 2001	No	N/A	Yes	Yes	Yes	Yes	Yes	Yes	Yes	Yes	9	Low Risk
79	A survey of insecticide resistance in *Aedes aelbopictus* (Diptera ; Culicidae) During a 2014 Dengue Fever outbreak in Guangzhou, China	Wang Yiguan et.al, 2017	No	Yes	Yes	Yes	Yes	Yes	Yes	Yes	Yes	Yes	9	Low Risk
80	Correlation between adult pyrethroid resistance and knockdown resistance (kdr) mutations in Aedes albopictus (Diptera: Culicidae) field populations in China	Jing-Peng Gao et.al, 2018	No	Yes	Yes	Yes	Yes	Yes	Yes	Yes	Yes	Yes	9	Low Risk
81	Evidence for multiple-insecticide resistance in urban Aedes albopictus populations in southern China	Yiji Li et.al, 2018	No	Yes	Yes	Yes	Yes	Yes	Yes	Yes	Yes	No	8	Moderate Risk
82	Insecticide Resistance of *Aedes albopictus* in Zhejiang Province, China	Juan Hou et.al, 2020	No	Yes	Yes	Yes	Yes	Yes	Yes	Yes	Yes	Yes	9	Low Risk
83	Monitoring Insecticide Resistance and Target Site Mutations of L1014 Kdr And G119 Ace Alleles in Five Mosquito Populations in Korea	Seo Hye Park et.al, 2020	No	Yes	Yes	Yes	Yes	Yes	Yes	Yes	Yes	No	8	Moderate Risk
84	Adulticide Resistance Status of *Aedes albopictus* (Diptera: Culicidae) in Sarawak State, Malaysia	Koon Weng Lau et.al, 2021	No	Yes	Yes	Yes	Yes	Yes	Yes	Yes	Yes	No	8	Moderate Risk
85	*Aedes Albopictus* (Diptera: Culicidae) Susceptibility Status to Agrochemical Insecticides Used in Durian Planting Systems in Southern Thailand	Sakda Ratisupakorn et.al, 2021	Yes	Yes	Yes	Yes	Yes	Yes	Yes	Yes	Yes	No	9	Low Risk

86	Assessment of the susceptibility status of *Aedes albopictus* (Diptera: Culicidae) from Interior, Sandakan and Tawau divisions of Sabah, Malaysia based on WHO diagnostic doses of larvicides	Elia-Amira, N.M.R et.al, 2021	No	Yes	Yes	Yes	Yes	Yes	Yes	Yes	Yes	No	8	Moderate Risk
87	Insecticide Susceptibility Status of *Aedes albopictus* in Dengue Hotspot Areas in Malaysia	Wan Fatma Zuharah et.al, 2021	No	Yes	Yes	Yes	Yes	Yes	Yes	Yes	Yes	No	8	Moderate Risk
88	The discovery of a novel knockdown resistance (*kdr*) mutation A1007G on *Aedes aegypti* (Diptera: Culicidae) from Malaysia	Wan Fatma Zuharah et.al, 2021	No	Yes	Yes	Yes	Yes	Yes	Yes	Yes	Yes	Yes	9	Low Risk
89	Insecticide susceptibility status and knockdown resistance (kdr) mutation in Aedes albopictus in China	Yong Wei et.al, 2021	Yes	Yes	Yes	Yes	Yes	Yes	Yes	Yes	Yes	Yes	10	Low Risk
90	First report of V1016G and S989P knockdown resistant (kdr) mutations in pyrethroid-resistant Sri Lankan Aedes aegypti mosquitoes	Sachini D. Fernando1 et.al, 2018	No	Yes	Yes	Yes	Yes	Yes	Yes	Yes	Yes	Yes	9	Low Risk
91	Knockdown Resistance (kdr) Mutations I1532T and F1534S Were Identified in Aedes albopictus Field Populations in Zhejiang Province, Central China	Yuyan Wu et.al, 2021	No	Yes	Yes	Yes	Yes	Yes	Yes	Yes	Yes	No	8	Moderate Risk
92	Multiple insecticide resistance and associated mechanisms to volatile pyrethroid in an Aedes albopictus population collected in southern China	Xiaodong Yang et.al, 2021	No	Yes	Yes	Yes	Yes	Yes	Yes	Yes	Yes	No	8	Moderate Risk
93	Widespread Distribution of a Newly Found Point Mutation in Voltage-Gated Sodium Channel in Pyrethroid-Resistant Aedes aegypti Populations in Vietnam	H. Kawada et.al, 2009	Yes	Yes	Yes	Yes	Yes	Yes	Yes	Yes	Yes	No	9	Low Risk
94	Voltage-gated sodium channel intron polymorphism and four mutations comprise six haplotypes in an Aedes aegypti population in Taiwan	Han-Hsuan Chung et.al, 2019	Yes	Yes	Yes	Yes	Yes	Yes	Yes	Yes	Yes	Yes	10	Low Risk
