# Supplementary material for: Updating the Insecticide Resistance Status of Aedes aegypti and Aedes albopictus in Asia: A Systematic Review and Meta-Analysis
Source: Trop Med Infect Dis. 2022 Oct 17;7(10):306. doi: 10.3390/tropicalmed7100306 (PMC9607256; doi:10.3390/tropicalmed7100306)
Supplement: Supplementary file 1 [file tropicalmed-07-00306-s001.zip › Table S4.pdf]

Table S4. Knockdown resistance (kdr) mutations that have been detected in different mosquito populations in Asia Region.

Country	*Point mutation and species*	*References*
Indonesia	*Ae. aegypti* (T506T, F1534C, V1016G, S989P)	1-6
Thailand	*Ae. aegypti* (V1016G, S989P, I1011V, V1016L, F1534C)	7-11
Malaysia	*Ae. aegypti* (F1534C, V1016G, S989P)	12
Laos	*Ae. aegypti* (F1534C,V1016G)	13
Vietnam	*Ae. aegypti* (V1016G, F1269C)	14
India	*Ae. aegypti* (T1520I, F1534C, V1016G, S989P)	15-17
Sri Lanka	*Ae. aegypti* (F1534C, V1016G, S989P)	18
China	*Ae. albopictus* (F1534S, F1534L, V1016G, I1532T)	19
Taiwan	*Ae. albopictus* (F1534C, V1016G, S989P, D1763Y)	20

References:

1. Hamid, P. H., Prastowo, J., Ghiffari, A., Taubert, A., and Hermosilla, C. 2017. "*Aedes aegypti* resistance development to commonly used insecticides in Jakarta, Indonesia," PLoS ONE, vol. 12, no. 12.
2. Hamid PH, Prastowo J, Ghifari A, Taubert A, Hermosilla C. 2017. *Aedes aegypti* resistance development to commonly used insecticides in Jakarta, Indonesia. PLoS ONE.;12(12):e0189680.
3. Hasmiwati, H., Rusjdi, S. and Nofita, E., 2018. Detection of Ace-1 gene with insecticides resistance in *Aedes aegypti* populations from DHF-endemic areas in Padang, Indonesia. Biodiversitas Journal of Biological Diversity, 19(1), pp.31-36.
4. Hamid, P., Ninditya, V., Prastowo, J., Haryanto, A., Taubert, A. and Hermosilla, C., 2018. Current Status of *Aedes aegypti* Insecticide Resistance Development from Banjarmasin, Kalimantan, Indonesia. BioMed Research International, pp.1-7.
5. Wuliandari, J., Hoffmann, A., Tantowijoyo, W. and Endersby-Harshman, N., 2020. Frequency of kdr mutations in the voltage-sensitive sodium channel (VSSC) gene in *Aedes aegypti* from Yogyakarta and implications for Wolbachia-infected mosquito trials. Parasites & Vectors, 13(1).
6. Hasmiwati, H. and Supargiyono, S., 2018. Short Communication: Genotyping of kdr allele in insecticide resistant-*Aedes aegypti* populations from West Sumatra, Indonesia. Biodiversitas Journal of Biological Diversity, 19(2), pp.502-508.
7. Stenhouse SA, Plernsub S, Yanola J, Lumjuan N, Dantrakool A, Choochote W, et al. 2013. Detection of the V1016G mutation in the voltagegated sodium channel gene of *Aedes aegypti* (Diptera: Culicidae) by allele-specifc PCR assay, and its distribution and efect on deltamethrin resistance in Thailand. Parasites Vectors;6(1):253.

8. Srisawat, R., Komalamisra, N., Eshita, Y., Zheng, M., Ono, K., Itoh, T., Matsumoto, A., Petmitr, S. and Rongsriyam, Y., 2010. Point mutations in domain II of the voltage-gated sodium channel gene in deltamethrin-resistant *Aedes aegypti* (Diptera: Culicidae). Applied Entomology and Zoology, 45(2), pp.275-282.

9. Yanola, J., Somboon, P., Walton, C., Nachaiwieng, W., Somwang, P. and Prapanthadara, L., 2011. High-throughput assays for detection of the F1534C mutation in the voltage-gated sodium channel gene in permethrin-resistant *Aedes aegypti* and the distribution of this mutation throughout Thailand. Tropical Medicine & International Health, 16(4), pp.501-509.

10. Rajatileka, S., Black, W., Saavedra-Rodriguez, K., Trongtokit, Y., Apiwathnasorn, C., McCall, P. and Ranson, H., 2008. Development and application of a simple colorimetric assay reveals widespread distribution of sodium channel mutations in Thai populations of *Aedes aegypti*. Acta Tropica, 108(1), pp.54-57.

11. Saingamsook, J., Saeung, A., Yanola, J., Lumjuan, N., Walton, C. and Somboon, P., 2017. A multiplex PCR for detection of knockdown resistance mutations, V1016G and F1534C, in pyrethroid-resistant *Aedes aegypti*. Parasites & Vectors, 10(1).

12. Leong, C., Vythilingam, I., Liew, J., Wong, M., Wan-Yusoff, W. and Lau, Y., 2019. Enzymatic and molecular characterization of insecticide resistance mechanisms in field populations of *Aedes aegypti* from Selangor, Malaysia. Parasites & Vectors, 12(1).

13. Tangena, J., Marcombe, S., Thammavong, P., Chonephetsarath, S., Somphong, B., Sayteng, K., Grandadam, M., Sutherland, I., Lindsay, S. and Brey, P., 2018. Bionomics and insecticide resistance of the arboviral vector Aedes albopictus in northern Lao PDR. *PLOS ONE*, 13(10), p.e0206387.

14. Kawada, H., Higa, Y., Komagata, O., Kasai, S., Tomita, T., Thi Yen, N., Loan, L., Sánchez, R. and Takagi, M., 2009. Widespread Distribution of a Newly Found Point Mutation in Voltage-Gated Sodium Channel in Pyrethroid-Resistant *Aedes aegypti* Populations in Vietnam. PLoS Neglected Tropical Diseases, 3(10), p.e527.

15. Kushwah, R. B. S., Dykes, C.L., Kapoor, N., Adak, T., and Singh, O. P. 2015. Pyrethroid- Resistance and Presence of Two Knockdown Resistance (kdr) Mutations, F1534C and a Novel Mutation T1520I, in Indian *Aedes aegypti*, PLOS Neglected Tropical Diseases, vol. 9, no. 1, p. e3332.

16. Saha, P., Chatterjee, M., Ballav, S., Chowdhury, A., Basu, N. and Maji, A., 2019. Prevalence of kdr mutations and insecticide susceptibility among natural population of *Aedes aegypti* in West Bengal. PLOS ONE, 14(4), p.e0215541.

17. Kushwah, R., Kaur, T., Dykes, C., Ravi Kumar, H., Kapoor, N. and Singh, O., 2020. A new knockdown resistance (kdr) mutation, F1534L, in the voltage-gated sodium channel of *Aedes aegypti*, co-occurring with F1534C, S989P and V1016G. Parasites & Vectors, 13(1).

18. Fernando, S., Hapugoda, M., Perera, R., Saavedra-Rodriguez, K., Black, W. and De Silva, N., 2018. First report of V1016G and S989P knockdown resistant (kdr) mutations in pyrethroid-resistant Sri Lankan *Aedes aegypti* mosquitoes. Parasites & Vectors, 11(1).

19. Zhou, X., Yang, C., Liu, N., Li, M., Tong, Y., Zeng, X. and Qiu, X., 2019. Knockdown resistance (kdr) mutations within seventeen field populations of Aedes albopictus from Beijing China: first report of a novel V1016G mutation and evolutionary origins of kdr haplotypes. Parasites & Vectors, 12(1).

20. Chung, H., Cheng, I., Chen, Y., Lin, C., Tomita, T. and Teng, H., 2019. Voltage-gated sodium channel intron polymorphism and four mutations comprise six haplotypes in an *Aedes aegypti* population in Taiwan. PLOS Neglected Tropical Diseases, 13(3), p.e0007291.
