# Supplementary material for: Updating the Insecticide Resistance Status of Aedes aegypti and Aedes albopictus in Asia: A Systematic Review and Meta-Analysis
Source: Trop Med Infect Dis. 2022 Oct 17;7(10):306. doi: 10.3390/tropicalmed7100306 (PMC9607256; doi:10.3390/tropicalmed7100306)
Supplement: Supplementary file 1 [file tropicalmed-07-00306-s001.zip › Table S5-S10.pdf]

Table S5. Meta-regression based on countries (*Ae. aegypti*). (compared group: Thailand).

Mixed-effect, Z distribution						
	Point estimate	Standard error	95% Lower	95% Upper	*z-value*	*p-value*
**Intercept**	-0.7292	0.2370	-1.1937	-0.2648	-3.0772	0.0021*
Cambodia	1.0385	0.5960	-0.1296	2.2066	1.7424	0.0814
India	-0.6796	0.3947	-1.4523	0.0941	-1.7216	0.0851
Indonesia	0.2816	0.3952	-0.4931	1.0562	0.7124	0.4762
Malaysia	0.8989	0.3301	0.2519	1.5459	2.7232	0.0065*
Pakistan	-0.9041	0.9755	-2.8161	1.0078	-0.9268	0.3540
Singapore	0.7600	0.5945	-0.4053	1.9252	1.2783	0.2012
Sri Lanka	0.2568	0.5297	-0.7815	1.2951	0.4848	0.6278
Vietnam	1.2687	0.6021	0.0886	2.4489	2.1071	0.0351*

* p-value <0.05.
With a test of Moderators (Coefficients 2:9) ;.
(df=8) = 25.2168, *p-value* = 0.0014*.

Table S6. Meta-regression based on countries (*Ae. albopictus*). (compared group: Thailand).

Mixed-effect, Z distribution						
	Point estimate	Standard error	95% Lower	95% Upper	*z-value*	*p-value*
**Intercept**	-3.6293	0.6932	-4.9878	-2.2707	-5.2358	0.0001*
China	1.9568	1.0023	-0.0076	3.9212	1.9523	0.0509
India	0.7239	0.9351	-1.1088	2.5566	0.7741	0.4388
Laos	2.3121	1.7600	-1.1375	5.7617	1.3137	0.1890
Malaysia	2.6165	0.7972	1.0541	4.1788	3.2823	0.0010*
Pakistan	1.8475	1.7610	-1.6041	5.2990	1.0491	0.2941
Singapore	2.8353	1.7597	-0.6137	6.2842	1.6112	0.1071
Sri Lanka	3.3187	1.3388	0.6948	5.9426	2.4789	0.0132*

* p-value <0.05.
With a test of Moderators (Coefficients 2:8) ;.
(df=7) = 16.1588, *p-value* = 0.0237*.

Table S7. Meta-regression based on Human Development Index (HDI) (*Ae. aegypti*).

Mixed-effect, Z distribution						
	Point estimate	Standard error	95% Lower	95% Upper	*z-value*	*p-value*
**Intercept**	-3.1950	1.1186	-5.3875	-1.0026	-2.8562	0.0043*
HDI	3.8716	1.5423	0.8488	6.8944	2.5104	0.0121*

* p-value <0.05.
With a test of Moderators (Coefficients 2);.
(df=1) = 6.3019, *p-value* = 0.0121*.

Table S8. Meta-regression based on Human Development Index (HDI) (*Ae. albopictus).*

Mixed-effect, Z distribution						
	Point estimate	Standard error	95% Lower	95% Upper	*z-value*	*p-value*
**Intercept**	-5.7622	2.7937	-11.2377	-0.2867	-2.0626	0.0392*
HDI	5.1930	3.7526	-2.1619	12.5479	1.3839	0.1664

* p-value <0.05.
With a test of Moderators (Coefficients 2);.
(df=1) = 1.9150, *p-value* = 0.1664.

Table S9. Meta-regression based on study quality (*Ae. aegypti*).

Mixed-effect, Z distribution						
	Point estimate	Standard error	95% Lower	95% Upper	*z-value*	*p-value*
**Intercept**	-0.8316	0.4328	-1.6799	0.0167	-1.9214	0.0547
Study quality	0.2848	0.2750	-0.2542	0.8238	1.0358	0.3003

* p-value <0.05.
With a test of Moderators (Coefficients 2);.
(df=1) = 1.0728, *p-value* = 0.3003.

Table S10. Meta-regression based on study quality (Ae. albopictus).

Mixed-effect, Z distribution						
	Point estimate	Standard error	95% Lower	95% Upper	*z-value*	*p-value*
**Intercept**	-1.9829	0.4382	-2.8418	-1.1240	-4.5249	0.0001*
Study quality	0.1218	0.6605	-1.1729	-1.4164	0.1844	0.8537

* p-value <0.05.
With a test of Moderators (Coefficients 2);.
(df=1) = 0.0340, *p-value* = 0.8537.
