# Supplementary figures and images for: Updating the Insecticide Resistance Status of Aedes aegypti and Aedes albopictus in Asia: A Systematic Review and Meta-Analysis
Source: Trop Med Infect Dis. 2022 Oct 17;7(10):306. doi: 10.3390/tropicalmed7100306 (PMC9607256; doi:10.3390/tropicalmed7100306)

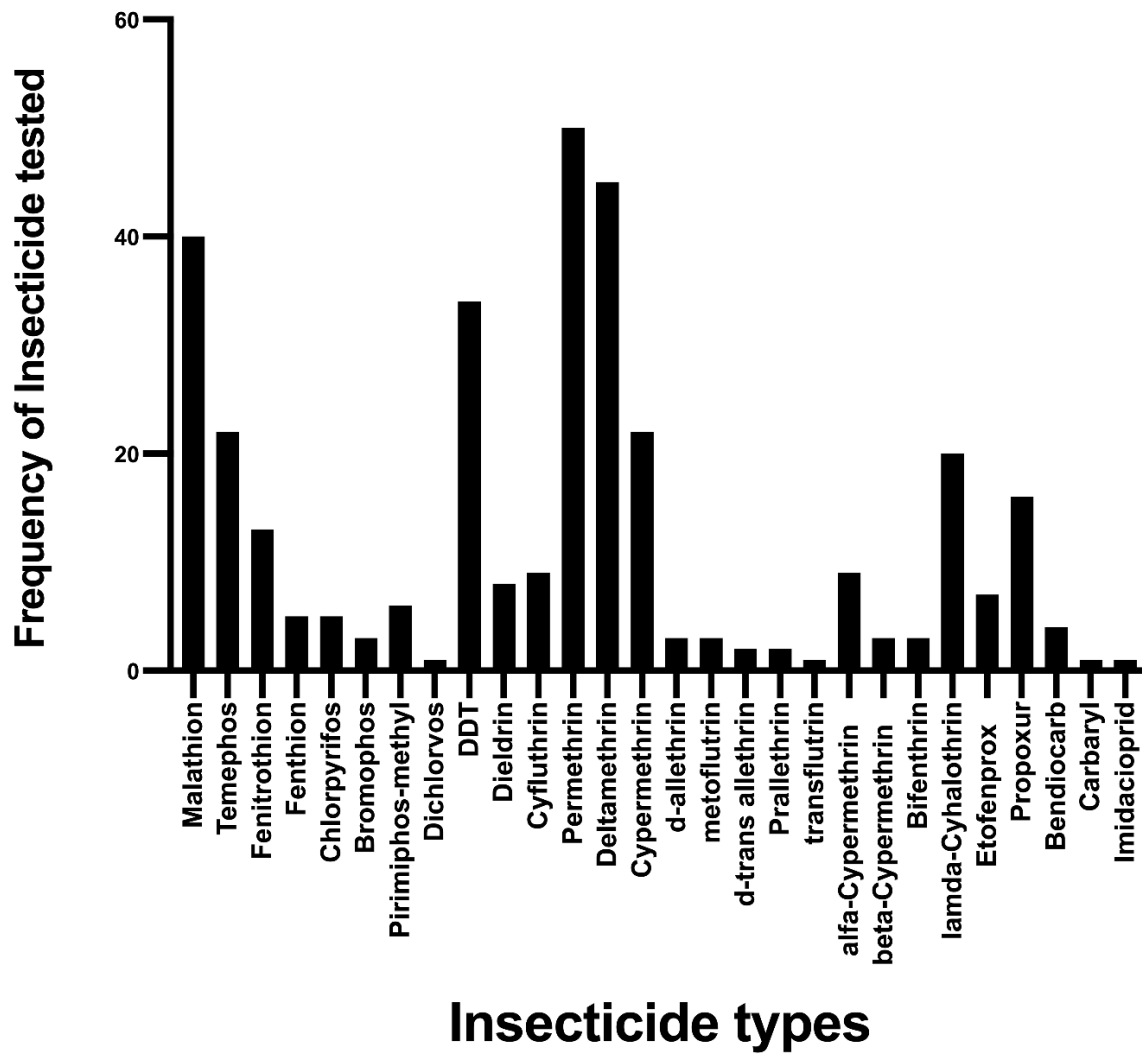

**Figure S1.** The frequency of insecticide resistance tested in the eligible articles.

Supplement: Supplementary file 1 [file tropicalmed-07-00306-s001.zip › Figure S1.pdf]
